# Effects of Dietary Butyrate Supplementation on Digestive Physiology, Feed Utilization, and Growth Performance in Crustaceans: A Meta-Analysis and Meta-Regression

**DOI:** 10.3390/ani16142186

**Published:** 2026-07-14

**Authors:** Moussa Gouife, Tchouli Noufeu, Tinghong Ming, Fei Kong, Lefei Jiao, Jiajie Xu

**Affiliations:** 1 Microbial Development and Metabolic Engineering Laboratory, School of Marine Science, Ningbo University, Ningbo 315211, China; gouife@nbu.edu.cn (M.G.);; 2 National Advanced School of Maritime and Ocean Science and Technology (ENSTMO), University of Ebolowa, Kribi P.O. Box 292, Cameroon; 3 National Higher School of Agriculture, Fisheries and Veterinary Medicine (ENSAHV), University of Douala, Douala P.O. Box 7236, Cameroon

**Keywords:** butyrate, crustacean aquaculture, feed additives, feed efficiency, digestibility, productivity, meta-analysis

## Abstract

Butyrate, a short-chain fatty acid (SCFA), is increasingly recognized as a functional feed additive in aquaculture. However, its effects on crustacean health and performance remain unclear. This meta-analysis synthesizes findings from 23 studies (2011–2025) examining butyrate supplementation across four crustacean groups: shrimp, crabs, crayfish, and prawns. Effect sizes were calculated for growth performance (final body weight, specific growth rate, survival rate), feed efficiency (feed conversion ratio, protein efficiency ratio), and digestive enzyme activities (amylase, lipase, alkaline phosphatase, total protease) in freshwater and marine environments. Results indicate that butyrate supplementation generally enhanced the digestive enzyme activity in both freshwater and marine environments, although the magnitude of the effect varied among enzyme types and habitats, with freshwater species showing improvements in alkaline phosphatase, amylase, lipase, and total protease, and marine species showing improvements in amylase, lipase, and total protease. Feed efficiency improved in freshwater (protein efficiency ratio) and marine species (feed conversion ratio). Growth-related outcomes, including final body weight and survival rate, were generally improved across both environments. Dose–response analyses indicated that dietary inclusion levels of 1–2% were associated with the most consistent improvements in digestive enzyme activity and growth performance. These findings support the potential use of butyrate as a functional feed strategy to improve productivity and sustainability in crustacean aquaculture. These effects may be mediated by multiple mechanisms, including improved intestinal epithelial integrity, the modulation of gut microbiota, and the regulation of immune and metabolic processes.

## Simple Summary

Crustacean farming is an important source of food worldwide, but farmers face challenges in keeping their animals healthy and growing efficiently. Butyrate, a natural compound produced by the fermentation of dietary fiber, has shown promise as a feed additive to improve animal health and growth. However, its effects on different crustacean species and under different conditions have not been clearly established. In this study, we synthesized results from 23 scientific experiments conducted between 2011 and 2025 to determine whether butyrate supplementation benefits crustacean aquaculture. Our analysis indicates that dietary butyrate supplementation generally improves digestive capacity, growth-related performance, and survival across several crustacean groups, although responses vary by species, habitat, and experimental conditions. The most consistent improvements in digestive enzyme activity and growth performance were observed at dietary inclusion levels of 1–2%. This study provides quantitative evidence supporting butyrate supplementation as a promising strategy for improving productivity and sustainability in crustacean farming.

## 1. Introduction

Aquaculture has emerged as a cornerstone of global food production, providing a sustainable and efficient source of protein to meet the growing demand for seafood [1,2]. The sector has experienced rapid expansion, driven by an increasing global population and the need to alleviate pressure on wild fish stocks [3,4,5]. Among the various aquaculture industries, crustacean farming, which includes shrimp, crabs, and prawns, has experienced the most substantial growth, particularly over the past few decades. Crustaceans now constitute a significant portion of global seafood production, with annual production increasing by approximately 10% from 2000 to 2018 [6,7]. However, this growth poses significant challenges to maintaining sustainability and improving productivity, particularly in optimizing growth rates, feed efficiency, and digestive health, while minimizing environmental impacts [8,9]. Traditional approaches addressing these challenges have relied on synthetic chemical additives, including antibiotic growth promoters, chemotherapeutic agents, and other functional additives, which may raise concerns about antimicrobial resistance, environmental sustainability, and long-term ecological impacts. In contrast, recent advancements have highlighted butyrate supplementation as a promising natural alternative [10,11].

Butyrate, a short-chain fatty acid (SCFA), has shown potential for enhancing growth rates, feed conversion efficiency, and immune responses in crustaceans by promoting gut health and stimulating the activity of digestive enzymes such as amylase and lipase [12]. It has also been found to improve intestinal morphology, an essential factor for maintaining the health and productivity of farmed crustaceans [13]. Due to its natural compatibility with metabolic pathways, butyrate can be safely incorporated into aquafeed formulations without significant ecological risk [14]. Despite the promising benefits of butyrate supplementation in aquaculture, its effectiveness remains unclear, and its impact can vary by species, environmental conditions, and dosage.

Crustacean species exhibit distinct responses to butyrate, driven by differences in their digestive systems, metabolic pathways, and habitat-specific factors. For instance, marine species such as the mud crab (*Scylla paramamosain*) are exposed to higher salinity, which may influence metabolic pathways and enzyme activities, potentially enhancing the effects of butyrate by modifying the gut microbiota composition and enzyme production [15]. On the other hand, freshwater species such as the narrow-clawed crayfish (*Astacus leptodactylus leptodactylus*), which inhabit environments with lower ionic strength, may experience distinct microbiome interactions that can affect the bioavailability and efficacy of butyrate [16]. These species-specific metabolic differences suggest that habitat plays a crucial role in determining the outcomes of butyrate supplementation, underscoring the need for tailored feed formulations.

Despite its potential, research on butyrate supplementation in aquaculture has yielded inconsistent results. For example, studies on Pacific white shrimp (*Litopenaeus vannamei*) show mixed outcomes, with some indicating significant improvements in digestive enzyme activity, FBW, FCR, and SUR [17,18,19]. However, other studies have found minimal or no significant effects, even when similar dosages were tested [20,21]. Furthermore, the optimal butyrate dosage remains unclear, as various studies have tested different concentrations with conflicting outcomes. For instance, an experiment with ridgetail white prawn (*Exopalaemon carinicauda*) found that supplementation with 0.5% butyrate led to significant improvements in growth performance, while higher dosages provided no additional benefit [22]. In contrast, studies on the Chinese mitten crab (*Eriocheir sinensis*) have found that higher butyrate concentrations (1–2%) improve the survival and growth metrics [23]. However, direct comparisons between these studies should be made with caution, as they differed in experimental duration, developmental stage, and species-specific physiological characteristics. These differences may partly explain the divergent dose–response patterns observed between species.

This study seeks to address these knowledge gaps through a comprehensive meta-analysis of studies published between 2011 and 2025, focusing on four major crustacean species: shrimp, crabs, prawns, and crayfish. By systematically evaluating the effects of butyrate supplementation on key indices of digestive health (alkaline phosphatase (ALP), amylase (AMY), lipase (LIP), and total protease (TP)), feed efficiency (feed conversion ratio (FCR) and protein efficiency ratio (PER)), and growth performance (final body weight (FBW), survival rate (SR), and specific growth rate (SGR)), this study aims to answer two critical research questions: (i) How does butyrate supplementation affect these biological outcomes in freshwater vs. marine crustacean species? and (ii) What is the optimal dosage of butyrate for enhancing digestive enzyme activity, feed efficiency, and growth performance while minimizing adverse effects? By identifying consistent patterns and examining how dosage and environmental conditions affect these outcomes, this meta-analysis will provide insights into the most effective methods for implementing butyrate supplementation in crustacean aquaculture. Ultimately, this research provides evidence-based recommendations for enhancing productivity and sustainability in crustacean farming, thereby reducing the reliance on antibiotics and other unsustainable practices.

## 2. Materials and Methods

### 2.1. Study Selection

A comprehensive literature search was conducted to identify studies evaluating the effects of butyrate supplementation on the growth, feed efficiency, and digestive health in crustaceans. The following electronic databases were searched: Web of Science, Scopus, and AGRIS using predefined search terms. These terms included (“butyrate*” OR “short-chain fatty acid*” OR “SCFA*” OR “organic acid*”) AND (“crustacean*” OR “shrimp*” OR “prawn*” OR “crab*” OR “lobster*”) AND (“growth*” OR “performance*” OR “efficiency*” OR “digestive enzymes*”) AND (“diet*” OR “dietary*” OR “feed*” OR “aquafeed*”). The search was limited to studies published in English. This systematic review and meta-analysis was conducted and reported in accordance with the Preferred Reporting Items for Systematic Reviews and Meta-Analyses (PRISMA) guidelines.

**Figure 1:**
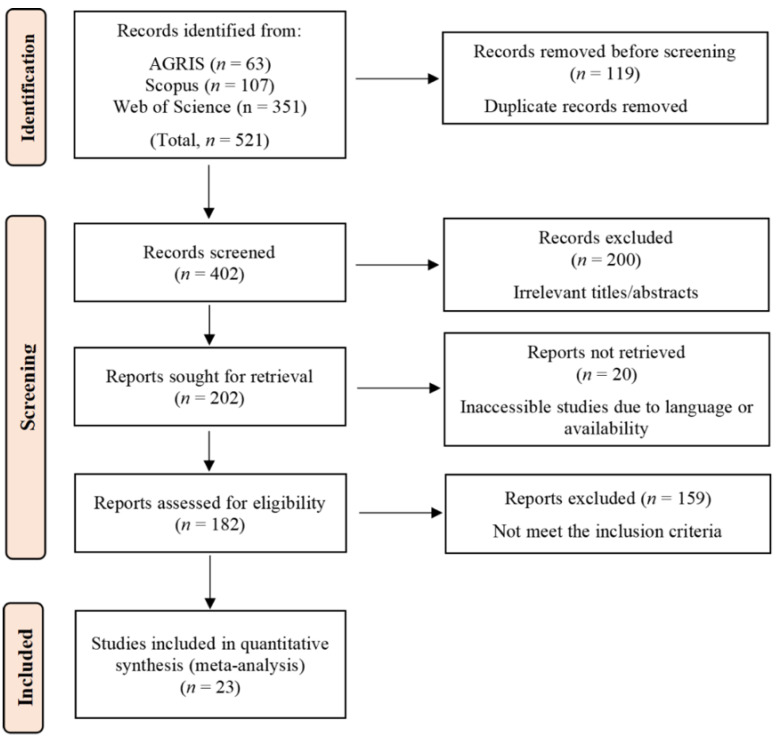
PRISMA workflow for study inclusion.

The inclusion criteria required that studies focus on crustaceans (shrimp, prawns, crabs, lobsters), involve supplementation with butyrate or SCFAs, and report measurable outcomes related to growth performance (FBW, SGR, SR), feed efficiency (FCR, PER), or digestive health (ALP, AMY, LIP, TP). Studies were excluded if they: (1) lacked sufficient quantitative data for effect size calculations or did not report the primary outcomes; (2) were non-experimental; (3) lacked appropriate control groups or contained unresolvable confounding factors; (4) focused on non-crustacean species or did not directly evaluate butyrate supplementation; or (5) evaluated butyrate only as part of a combined formulation with other functional additives (e.g., probiotics or organic acid blends) without a separate treatment group allowing the independent effect of butyrate to be assessed. Ultimately, 23 studies published between 2011 and 2025 were selected for inclusion in the meta-analysis (Figure 1).

### 2.2. Data Extraction

Data were systematically extracted from each study based on key growth performance indicators, feed efficiency indices, and digestive health parameters. Additionally, experimental variables were extracted to provide context, including species, developmental stage, habitat type, and concentration and delivery method of butyrate supplementation. The formulation type of butyrate supplementation was also extracted whenever available, including free butyric acid, sodium butyrate, coated or microencapsulated sodium butyrate. These formulation characteristics were considered relevant because differences in physicochemical properties, stability during feed processing, and gastrointestinal release profiles may influence biological responses. Environmental factors, including water quality parameters, stocking density, and feeding rate, were also recorded, as these can influence the study outcomes. The experimental duration, acclimation period, feeding frequency, number of experimental groups, and replicates were also documented (Table S1). For studies presenting graphical data, numerical values were extracted using the WebPlotDigitizer (version 4.8), which was required for 6 out of the 23 included studies (26%); for the remaining studies, numerical values were directly extracted from the text or tables. Any missing data were sought from the authors to ensure consistency. The extracted data were organized into a standardized table that included study-specific details to ensure accuracy and transparency for subsequent analysis.

### 2.3. Statistical Analysis

All statistical analyses were performed using R software (version 4.4.1). For the primary analysis, we employed meta-analysis to assess the overall effect of butyrate supplementation on crustacean aquaculture performance. Specifically, standardized mean differences (SMD) and treatment effects (TE) across studies were calculated using Hedges’ g (effect size), as previously described [24,25]. This method was chosen for its ability to correct for small-sample-size biases and provide a standardized measure of the effect of butyrate supplementation. The effect sizes were derived from the means and standard deviations of each study. For feed efficiency outcomes, FCR and PER were interpreted according to their biological meaning: lower FCR and higher PER indicate improved feed efficiency. Given their different numerical directions, pooled estimates combining these indicators were interpreted cautiously. A random-effects model was used to account for variability in true effect sizes across studies, with the between-study variance (τ^2^) estimated using the restricted maximum likelihood (REML) method.

Heterogeneity across studies was assessed using the I^2^ statistic, which quantifies the proportion of total variation in effect estimates due to between-study variability. A high I^2^ value (above 50%) indicates substantial heterogeneity, suggesting that the studies are not measuring the same effect [26]. We conducted subgroup analyses based on key variables, including habitat type (marine, freshwater) and butyrate concentration, to further explore potential sources of heterogeneity. Taxonomic subgroup analyses were considered to evaluate potential differences among shrimp, prawns, crayfish, and crabs; however, these analyses were not performed systematically because the number of studies available within each taxonomic group was insufficient to provide robust comparative estimates. These subgroup analyses allowed us to assess whether the effect of butyrate supplementation varied across different study characteristics.

We assessed publication bias using several methods. First, we examined funnel plots to visually detect asymmetry, which could suggest publication bias. Egger’s test was also performed to statistically assess funnel plot asymmetry, with significant asymmetry indicating potential bias [27]. Additionally, we applied the trim-and-fill method to adjust for publication bias by imputing missing studies, thus recalculating the overall effect size and providing a bias-adjusted estimate of the treatment effect [28].

Finally, to investigate the relationship between butyrate application rates and biological outcomes, we performed a meta-regression using the metafor package (version 4.6-0) in R. This analysis assessed whether varying butyrate doses affected the observed effect sizes. Meta-regression was conducted using a univariate model, with butyrate dose as the moderator and effect size (Hedges’ g) as the dependent variable. The results of these analyses were visualized with plots illustrating the dose–response relationship, providing further insight into how the butyrate application rates affect aquaculture outcomes.

## 3. Results

### 3.1. Study Selection and Overview of Dataset

A comprehensive literature search was conducted across multiple databases, including Web of Science, Scopus, and AGRIS, using specific search terms related to butyrate supplementation and crustacean species. The initial search identified 521 records, and after removing 119 duplicates, 402 records were screened. Of these, 200 were excluded due to irrelevant titles or abstracts. From the remaining 202 records sought for retrieval, 20 could not be retrieved due to language or availability issues. This left 182 reports to be assessed for eligibility. Of these, 159 were excluded for failing to meet the inclusion criteria. Ultimately, 23 published studies were selected for inclusion in the meta-analysis (Figure 1). These studies, conducted between 2011 and 2025, investigated the effects of butyrate supplementation across four major crustacean groups: crabs (3 species), shrimp (2 species), crayfish (2 species), and prawns (1 species), for a total of 8 species (Table S1). A detailed summary of the included studies, including species, habitat, butyrate formulation, dietary inclusion level, experimental duration, and evaluated response variables, is provided in Table S1. The included studies evaluated several butyrate formulations, including sodium butyrate, free butyric acid, coated or microencapsulated preparations. Due to the limited number of studies available for each individual formulation, all formulations were analyzed collectively in the meta-analysis. These taxonomic groups differ substantially in digestive morphology, nutrient utilization strategies, microbiota composition, and metabolic regulation, which may contribute to variability in their responses to dietary butyrate supplementation. The included studies covered several geographical regions, with a clear concentration in Asia. Specifically, 71% of the studies were conducted in Asian countries, while the remaining studies originated from South America, North America, Europe, and Oceania. This geographical distribution is further considered a limitation regarding the generalizability of the findings. Most studies focused on marine species, particularly *Litopenaeus vannamei* (Pacific white shrimp) and *Penaeus monodon* (black tiger shrimp). At the same time, a smaller subset examined freshwater species, including *Astacus leptodactylus* (narrow-clawed crayfish) and *Procambarus clarkii* (red swamp crayfish). These studies primarily focused on juvenile stages, although some included post-larvae and early juveniles. Experimental conditions varied widely, including differences in stocking densities, highlighting the diversity of farming systems. The duration of experiments ranged from 14 to 63 days, and feeding frequency ranged from 2 to 6 feedings per day, with most studies using 3 to 4 feedings per day. These variations underline the diversity in experimental protocols and the broad scope of research on butyrate supplementation in crustacean aquaculture.

### 3.2. Butyrate Effect on Digestive Enzymes

**Figure 2:**
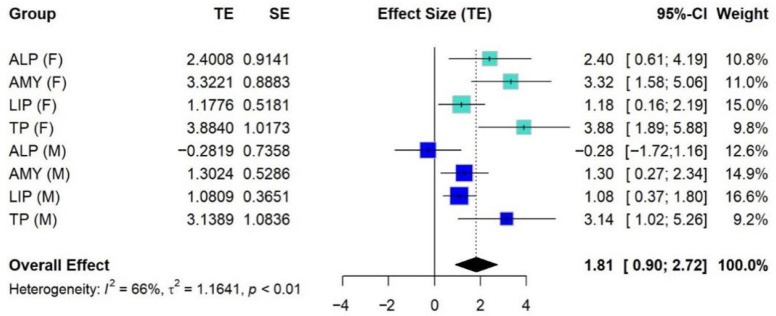
Forest plot of effect sizes (mean ± 95% CI) for digestive enzyme variables in freshwater and marine species supplemented with butyrate. Green squares represent freshwater species, whereas blue squares represent marine species. The vertical dashed line indicates the pooled overall effect estimate. ALP, alkaline phosphatase; AMY, amylase; CI, confidence interval (lower and upper); F, freshwater; I^2^, percentage variation across studies due to heterogeneity; LIP, lipase; M, marine; TP, total protease.

Butyrate supplementation significantly influenced digestive enzyme activity in freshwater and marine crustaceans, as indicated by the effect sizes and corresponding 95% confidence intervals (CIs) (Figure 2, Table 1). In freshwater crustaceans, ALP showed a significant positive effect with butyrate supplementation (ES = 2.4008; 95% CI = 0.6091, 4.1925; *p* = 0.0086). AMY also demonstrated a substantial impact (ES = 3.3221; 95% CI = 1.5810, 5.0631; *p* = 0.0002), as did LIP (ES = 1.1776; 95% CI = 0.1621, 2.1931; *p* = 0.023) and TP (ES = 3.8840; 95% CI = 1.8900, 5.8780; *p* = 0.0001). In marine environments, ALP had no significant effect (ES = −0.2819; 95% CI = −1.7242 to 1.1603; *p* = 0.7016). However, AMY showed a significant positive effect (ES = 1.3024; 95% CI = 0.2663, 2.3385; *p* = 0.0137), as did LIP (ES = 1.0809; 95% CI = 0.3654, 1.7964; *p* = 0.0031) and TP (ES = 3.1389; 95% CI = 1.0151, 5.2628; *p* = 0.0038). Butyrate supplementation significantly affected digestive enzymes across both environments (ES = 1.8099; 95% CI = 0.9002, 2.7195; *p* < 0.0001). These results highlight the potential of butyrate supplementation to improve digestive health in crustacean aquaculture, with notable differences between freshwater and marine settings.

### 3.3. Butyrate Effect on Feed Efficiency

**Figure 3:**
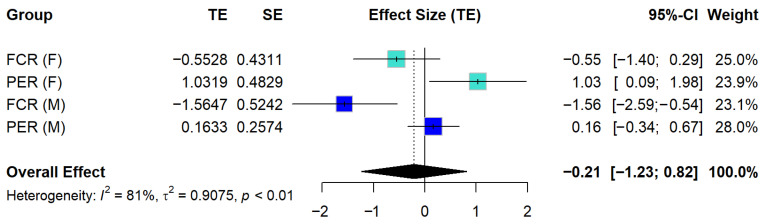
Forest plot of effect sizes (mean ± 95% CI) for feed efficiency variables in freshwater (F) and marine (M) species supplemented with butyrate. Green squares represent freshwater species, whereas blue squares represent marine species. The vertical dashed line indicates the pooled overall effect estimate. CI, confidence interval (lower and upper); F, freshwater; FCR, feed conversion ratio; I^2^, percentage variation across studies due to heterogeneity; M, marine; PER, protein efficiency ratio.

The analysis of feed-efficiency variables in freshwater and marine species, supplemented with butyrate, revealed distinct responses depending on the indicator considered (Figure 3, Table 1). In freshwater species, FCR showed a non-significant reduction (ES = −0.5528; 95% CI = −1.3977, 0.2922; *p* = 0.1998), whereas PER significantly improved (ES = 1.0319; 95% CI = 0.0855, 1.9784; *p* = 0.0326). In marine species, FCR was significantly reduced (ES = −1.5647; 95% CI = −2.5921, −0.5374; *p* = 0.0028), while PER showed no significant effect (ES = 0.1633; 95% CI = −0.3413, 0.6678; *p* = 0.5259). Because FCR and PER have opposite numerical directions, with lower FCR and higher PER values indicating improved efficiency, the pooled estimate combining these indicators should be interpreted cautiously. Significant heterogeneity was observed among feed efficiency outcomes (I^2^ = 81%), suggesting that responses varied across indicators and rearing environments. The overall pooled effect was not significant (ES = −0.2065; 95% CI = −1.2305, 0.8174; *p* = 0.6926); therefore, separate analyses provide a more biologically relevant interpretation, indicating potential improvements in FCR for marine species and PER for freshwater species.

### 3.4. Butyrate Effect on Growth Performance

**Figure 4:**
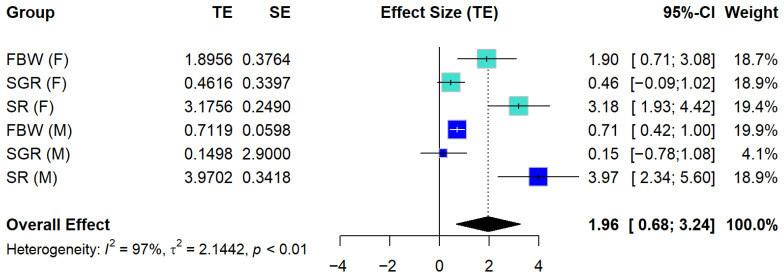
Forest plot of effect sizes (mean ± 95% CI) for growth performance variables in freshwater (F) and marine (M) species supplemented with butyrate. Green squares represent freshwater species, whereas blue squares represent marine species. The vertical dashed line indicates the pooled overall effect estimate. CI, confidence interval (lower and upper); F, freshwater; FBW, final body weight; I^2^, percentage variation across studies due to heterogeneity; M, marine; SGR, specific growth rate; SR, survival rate.

The analysis of growth performance variables in freshwater and marine species supplemented with butyrate revealed significant improvements in several indices (Figure 4, Table 1). For freshwater species, FBW showed a significant increase (ES = 1.8956; 95% CI = 0.7136, 3.0775; *p* = 0.0017), and SR also demonstrated a significant improvement (ES = 3.1756; 95% CI = 1.9304, 4.4207; *p* < 0.0001). However, SGR did not show a significant effect (ES = 0.4616; 95% CI: −0.0920 to 1.0153; *p* = 0.1022). In marine species, FBW increased significantly (ES = 0.7119; 95% CI = 0.4232, 1.0006; *p* < 0.0001), and SR improved significantly (ES = 3.9702; 95% CI = 2.3398, 5.6007; *p* < 0.0001). Conversely, SGR in marine species showed no significant effect (ES = 0.1498; 95% CI = −0.7782, 1.0778; *p* = 0.7517). The combined growth performance analysis indicated a significant positive effect (ES = 1.9583; 95% CI = 0.6759, 3.2407; *p* = 0.0028). These findings highlight the beneficial impact of butyrate supplementation on growth performance, with significant improvements in FBW and SR across freshwater and marine species.

### 3.5. Heterogeneity Analysis

The meta-analysis on the effects of butyrate supplementation in crustacean aquaculture revealed considerable variability across different variables (Table 1). For digestive enzymes, ALP in freshwater showed high heterogeneity (I^2^ = 81.6%), whereas AMY, LIP, and TP exhibited no heterogeneity. In marine environments, ALP demonstrated moderate heterogeneity (I^2^ = 57.2%), AMY showed substantial heterogeneity (I^2^ = 67%), LIP exhibited moderate heterogeneity (I^2^ = 53.2%), and TP showed considerable heterogeneity (I^2^ = 60.2%). Digestive enzyme activity indicated moderate heterogeneity (I^2^ = 66%) (Figure 2 and Figures S1–S8). Feed efficiency indices, including FCR and PER, showed no heterogeneity in freshwater. FCR in marine environments exhibited moderate heterogeneity (I^2^ = 37.5%), and PER showed no heterogeneity. Feed efficiency demonstrated substantial heterogeneity (I^2^ = 80.5%) (Figure 3 and Figures S9–S12). Regarding growth performance, FBW in freshwater showed substantial heterogeneity (I^2^ = 69.9%). In comparison, SGR and SR exhibited no heterogeneity and low heterogeneity (I^2^ = 19.1%), respectively. In marine settings, FBW demonstrated moderate heterogeneity (I^2^ = 23.7%), while SGR showed no heterogeneity, and SR exhibited high heterogeneity (I^2^ = 73.6%). Growth performance indices indicated substantial heterogeneity (I^2^ = 97.3%) (Figure 4 and Figures S13–S18). The observed heterogeneity is likely due to the interactions of multiple factors, including species differences, environmental conditions, and variations in experimental protocols. These factors contribute to the variability in the effects of butyrate supplementation on growth performance, feed efficiency, and digestive enzyme activity.

### 3.6. Publication Bias Assessment

**Figure 5:**
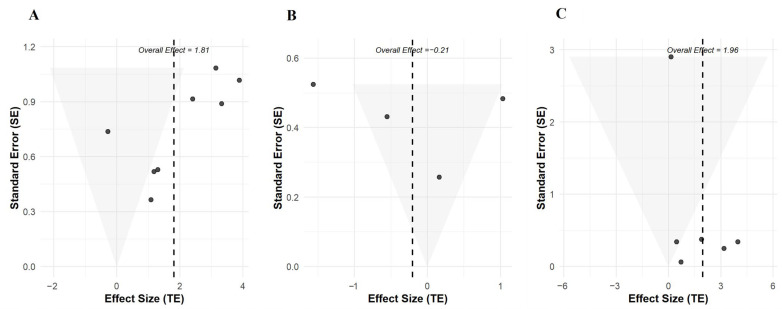
Funnel plots assessing publication bias for (**A**) digestive enzyme, (**B**) feed efficiency, and (**C**) growth performance. Each dot represents an individual study effect size, and the shaded triangle indicates the expected region of the funnel plot under no publication bias.

The publication bias assessment for the variables analyzed in this study was conducted using Egger’s test, with adjustments via the trim-and-fill method to assess the robustness of the findings (Figure 5 and Table 1). For digestive enzymes, significant publication bias was noted for ALP in freshwater (*p* = 0.002) and TP in both freshwater (*p* < 0.0001) and marine environments (*p* = 0.0001). Nevertheless, the positive effects on digestive enzyme activity indicate that butyrate supplementation enhances digestive health. Adjustments for bias still reflect a beneficial impact, albeit more conservatively estimated. Regarding feed efficiency, Egger’s test revealed no significant publication bias for FCR and PER in freshwater (*p* = 0.2028), but a significant bias was observed for FCR in marine environments (*p* = 0.0004). However, the overall positive effects on feed efficiency indices suggest that butyrate supplementation improves feed utilization, with adjustments providing a more conservative yet still positive estimate. For growth performance indices, Egger’s test indicated significant publication bias for FBW in freshwater (*p* < 0.0001) and SR in marine environments (*p* < 0.0001). In contrast, no significant bias was detected for SGR in both freshwater (*p* = 0.1022) and marine settings (*p* = 0.0939). Despite the detection of significant publication bias for FBW in freshwater and SR in marine environments, the overall effect sizes remained positive. Adjustments using the trim-and-fill method still indicate beneficial effects, suggesting that butyrate supplementation enhances these indices even after accounting for potential bias. In summary, the general results demonstrate a positive effect of butyrate supplementation on growth performance, feed efficiency, and digestive health in crustacean aquaculture. Adjustments for publication bias provide a more cautious interpretation but do not negate the overall beneficial impacts observed.

### 3.7. Analysis of Butyrate Application Rate

**Figure 6:**
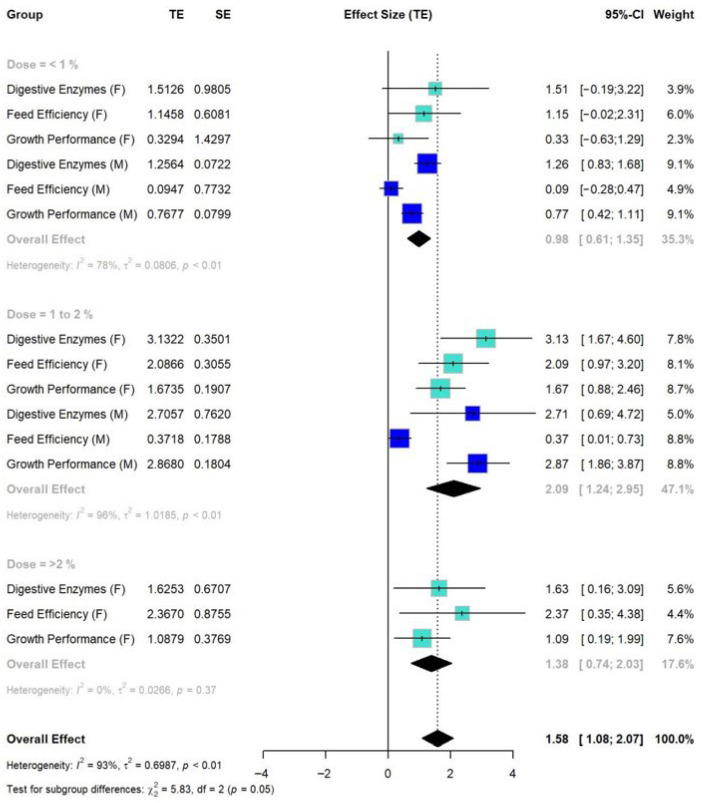
Forest plot of effect sizes for digestive enzymes, feed efficiency, and growth performance across different butyrate application rates in freshwater (F) and marine (M) species. Green squares represent freshwater species, whereas blue squares represent marine species. The vertical dashed line indicates the pooled overall effect estimate.

The forest plot illustrates the effect sizes of butyrate supplementation on digestive enzymes, feed efficiency, and growth performance across different application rates in freshwater and marine species (Figure 6). For doses less than 1%, butyrate supplementation showed a positive but non-significant effect on digestive enzymes in freshwater species (ES = 1.5126; 95% CI = −0.19, 3.22), whereas a significant positive effect was observed in marine species (ES = 1.2564; 95% CI = 0.83, 1.68), with overall heterogeneity (I^2^ = 78%, *p* < 0.01). Feed efficiency and growth performance in freshwater did not show significant effects (ES = 1.1458; 95% CI = −0.02, 2.31 and ES = 0.3294; 95% CI = −0.63, 1.29, respectively), whereas marine environments exhibited a significant positive effect on growth performance (ES = 0.7677; 95% CI = 0.42, 1.11) (Figure 6 and Figures S19–S24). For doses between 1% and 2%, significant positive effects were observed for digestive enzymes in both freshwater (ES = 3.1322; 95% CI = 1.67, 4.60) and marine environments (ES = 2.7057; 95% CI = 0.69, 4.72), with high overall heterogeneity (I^2^ = 98%, *p* < 0.01). Feed efficiency and growth performance also showed significant improvements in freshwater (ES = 2.0866; 95% CI = 0.97, 3.20 and ES = 1.6735; 95% CI = 0.88, 2.46, respectively) and marine environments (ES = 2.8680; 95% CI = 1.86, 3.87 for growth performance) (Figure 6 and Figures S25–S30). For doses greater than 2%, digestive enzymes in freshwater showed a significant effect (ES = 1.6253; 95% CI = 0.16, 3.09), while feed efficiency in freshwater also demonstrated a significant positive effect (ES = 2.3670; 95% CI = 0.35, 4.38), with no significant heterogeneity (I^2^ = 0%, *p* = 0.37) (Figure 6 and Figures S31–S33). Overall, the pooled analysis indicated a significant positive effect of butyrate supplementation across the evaluated indices and environments (ES = 1.58; 95% CI = 1.08, 2.07), although substantial heterogeneity was observed (I^2^ = 93%, *p* < 0.01). These findings underscore the dose-dependent benefits of butyrate supplementation on crustacean aquaculture, with notable variations between freshwater and marine settings. Based on these findings, butyrate supplementation at 1–2% generally produced the most consistent positive responses in digestive enzymes, feed efficiency, and growth performance across freshwater and marine environments, suggesting this range as a potentially effective inclusion level for crustacean aquaculture.

### 3.8. Meta-Regression Analysis

**Figure 7:**
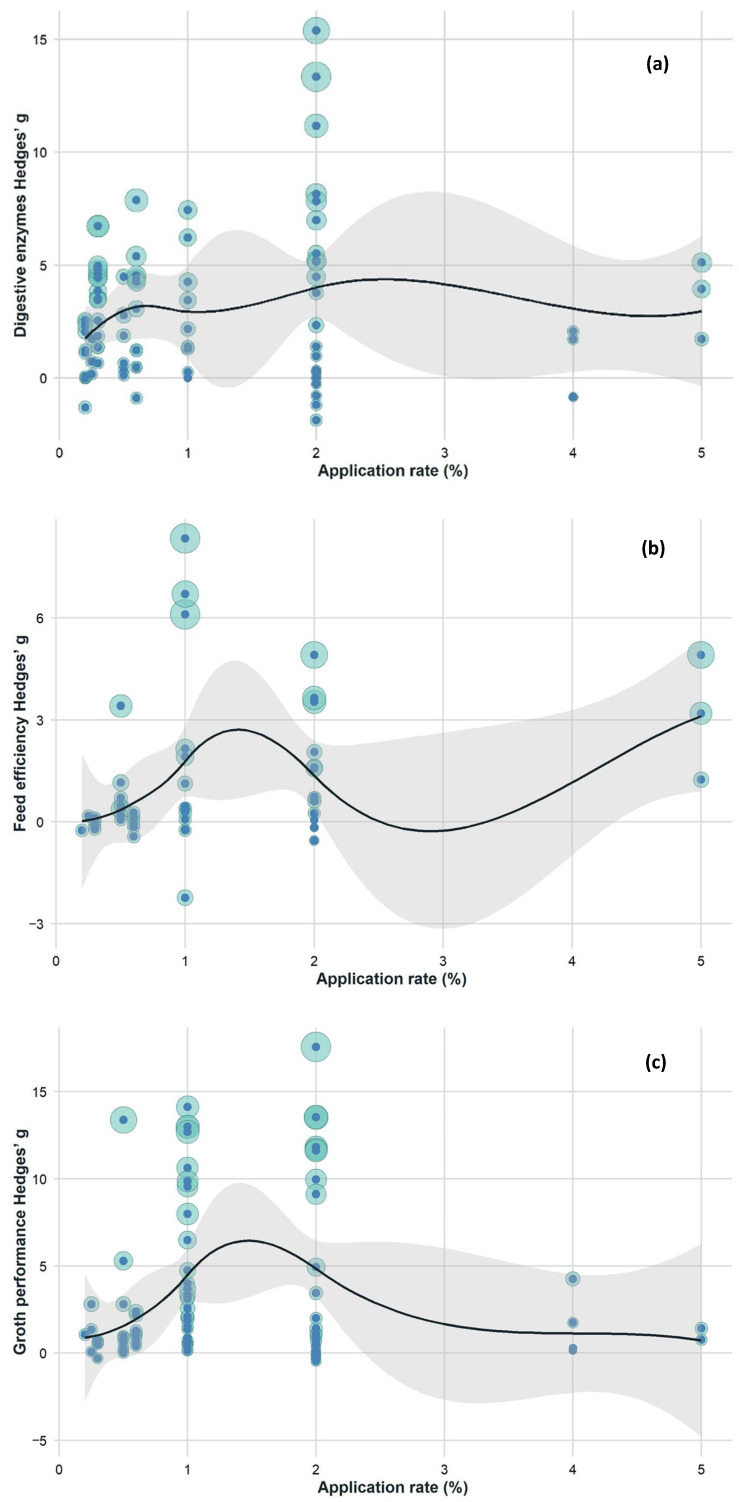
Meta-regression analysis of butyrate application rate on (**a**) digestive enzymes, (**b**) feed efficiency, and (**c**) growth performance. The circle center and diameter represent the mean and 95% confidence interval of the effect size.

The meta-regression analysis examines the relationship between the application rate of butyrate and its effects on digestive enzymes, feed efficiency, and growth performance in crustacean aquaculture (Figure 7). The baseline effect, as determined by the intercept, indicated a significant positive impact on digestive enzymes (Intercept = 1.6500, *p* < 0.0001) and growth performance (Intercept = 0.9533, *p* < 0.0001), while feed efficiency showed no significant baseline effect (Intercept = −0.0186, *p* = 0.9256). The correlation with the application rate, as indicated by the slope, was not significant for digestive enzymes (Slope = 0.0287, *p* = 0.8761) and growth performance (Slope = −0.0353, *p* = 0.6945). However, it was significant for feed efficiency (Slope = 0.3615, *p* = 0.0101). Residual heterogeneity, assessed using the QE statistic, revealed substantial heterogeneity for digestive enzymes (QE = 209.3381, *p* < 0.0001) and growth performance (QE = 219.6586, *p* < 0.0001). In contrast, feed efficiency did not exhibit significant residual heterogeneity (QE = 56.0930, *p* = 0.2898). These findings highlight a significant dose-dependent improvement in feed efficiency with butyrate supplementation, while digestive enzyme activity and growth performance do not exhibit significant dose–response relationships (Table 2).

## 4. Discussion

### 4.1. Impact of Butyrate Supplementation on Digestive Enzyme Activity

Butyrate supplementation significantly enhanced the digestive enzyme activity in both freshwater and marine environments, underscoring its potential to improve digestive health in crustacean aquaculture. Notably, the greatest numerical effect was observed on TP activity, which is crucial for breaking down dietary proteins into amino acids and peptides essential for growth. However, this result should be interpreted with caution, as TP was based on the fewest studies, showed evidence of publication bias, and exhibited the largest reduction in effect size after trim-and-fill adjustment. This enhancement in protease activity suggests that butyrate may help optimize nutrient digestion and absorption, improving the feed conversion efficiency and overall growth performance in crustaceans [29]. Beyond its effects on digestive enzymes, butyrate may improve crustacean physiological performance through multiple interconnected mechanisms. As a short-chain fatty acid, butyrate serves as an energy substrate for intestinal epithelial cells and may enhance intestinal barrier function through the regulation of tight junction proteins and mucus production. In addition, butyrate can modulate gut microbial communities by promoting beneficial microbial populations and altering microbial metabolite production. These effects may subsequently influence nutrient absorption, immune regulation, and metabolic homeostasis. Although these mechanisms are well-characterized in terrestrial animals and some fish species, their specific contribution in crustaceans remains insufficiently investigated and warrants further experimental validation. The positive results across freshwater and marine environments indicate that butyrate’s benefits are not limited to specific habitats, highlighting its broad applicability in diverse aquaculture settings. These findings align with existing research that suggests that butyrate plays a key role in improving gut health, metabolic efficiency, and nutrient utilization, all of which are vital for the sustainability and productivity of aquaculture systems [30,31,32]. Additionally, butyrate supplementation significantly increases AMY activity in freshwater and marine crustaceans, indicating its positive impact on starch digestion across aquatic environments. By improving AMY activity, butyrate optimizes the biochemical pathways that break down starches into simpler sugars, which are crucial for energy production and metabolism [33,34]. This enhancement supports more efficient nutrient assimilation, facilitating the crustaceans’ ability to convert dietary starches into usable energy for growth and development. Beyond its direct effect on digestive enzymes, butyrate also promotes the growth of beneficial gut microbes and reduces gut inflammation, further improving digestive health and boosting the organism’s resilience to stressors [35].

At butyrate supplementation levels ranging from 1% to 2%, significant positive effects have been observed on digestive enzymes in both freshwater and marine aquaculture environments. Specifically, these butyrate concentrations have been shown to enhance the activity of protease, lipase, and amylase, which are essential for efficient nutrient digestion and assimilation in aquatic species. This improvement in digestive enzyme activity results in enhanced feed efficiency and improved growth performance. Furthermore, similar doses are compatible with various fish species, making them applicable in both contexts [36,37,38,39,40]. However, the high heterogeneity (I^2^ = 81%, *p* < 0.01) observed across studies suggests substantial variability in the effects, indicating that important factors influencing the outcomes may not be fully accounted for. Although salinity, temperature, and nutrient load were not directly tested as moderators in this meta-analysis (only habitat type and dose were formally examined), these variables may represent potential sources of heterogeneity and are proposed here as hypotheses for future investigations rather than as data-supported explanations. For instance, salinity gradients may influence treatment responses in marine environments [41,42], while variations in nutrient availability and temperature conditions may modify physiological responses to supplementation [43,44]. Therefore, these environmental factors should be considered potential moderators and further validated in future studies.

### 4.2. Impact of Butyrate Supplementation on Feed Efficiency

Butyrate supplementation significantly improved PER in freshwater species, thereby enhancing protein utilization in these aquaculture systems. This improvement may be associated with butyrate-mediated effects on gut physiology, including the modulation of microbial communities and intestinal barrier function. These findings underscore butyrate’s capacity to enhance feed conversion efficiency and nutrient utilization by modulating gut microbiota composition and reinforcing intestinal barrier function, as demonstrated by several studies [45,46]. In marine species, butyrate significantly reduced FCR, indicating improved feed efficiency, by reducing the amount of feed required to gain a unit of body weight [10]. Lower FCRs indicate that less feed is needed to achieve comparable or superior growth outcomes, a finding with profound economic and ecological implications [47,48]. This highlights butyrate’s potential as a beneficial feed additive, promoting nutrient absorption and improving intestinal health in crustaceans, suggesting that enhanced metabolic efficiency may underlie this effect [16,49]. These findings are particularly valuable given the centrality of FCR in determining the sustainability of aquaculture operations; reducing FCR minimizes resource consumption and curtails waste production, mitigating environmental impacts [50].

The dose-dependent relationship between butyrate supplementation and feed efficiency in freshwater crustaceans showed a positive response across the entire evaluated dose range, including doses above 2%, with no clear plateau observed within the tested range. This pattern differed from digestive enzyme activity and growth performance, for which responses appeared to reach their highest levels at 1–2% inclusion. These differences suggest that the optimal dietary inclusion level may depend on the specific physiological parameter evaluated and the biological mechanisms underlying each response. However, a nuanced interpretation of the feed efficiency metrics FCR and PER is essential for fully understanding these results. Since FCR is significant when decreasing and PER when increasing, the pooled effect likely reflects the relative contribution of PER among the available feed efficiency outcomes, particularly in freshwater species where PER showed a significant response. This finding is consistent with prior analyses, which have demonstrated a significant effect of PER alone, whereas FCR did not show a significant effect in freshwater crustaceans. These insights underscore the need for further investigation into the differential contributions of these metrics to optimize feed efficiency in aquaculture.

The relatively low heterogeneity observed in our study, compared to previous meta-analyses of feed additive indices, can be partly attributed to the focused scope of our analysis. While other studies often pool data from a broad range of aquatic organisms, our meta-analysis specifically targeted crustaceans, further segmented into freshwater and marine species. This segmentation may reduce part of the variability associated with broad comparisons among unrelated aquatic taxa; however, substantial biological differences remain among crustacean groups, including differences in digestive physiology, microbiota composition, and metabolic strategies. For instance, studies on the role of β-glucan supplementation in finfish and shellfish exhibit extreme heterogeneity in FCR (I^2^ = 99.95%) [51]. Also, taurine supplementation in aquatic animals exhibits high heterogeneity in FCR (I^2^ = 98.77%) and PER (I^2^ = 99.12%) [52]. Similarly, supplementation with a methionine hydroxy analog in fish and shrimp shows considerable heterogeneity, with I^2^ values of 97.78% for FCR and 98.57% for PER [24]. In contrast, the consistent effects of butyrate on FCR and PER in freshwater environments, with no observed heterogeneity, suggest that species-specific or environmental factors may influence butyrate’s impact. However, the moderate heterogeneity observed for FCR in marine environments (I^2^ = 37.5%) may reflect environmental and experimental differences among studies, including variation in salinity, temperature, and dietary conditions, which should be evaluated as potential moderators. Our study’s more targeted approach allows for a clearer understanding of butyrate’s effects in crustacean aquaculture, suggesting that butyrate supplementation may be a promising intervention to improve feed efficiency, although responses remain influenced by species and environmental conditions.

### 4.3. Impact of Butyrate Supplementation on Growth Performance

The results of this meta-analysis indicate that butyrate supplementation is associated with improved growth performance and survival in freshwater and marine crustacean species. These effects may be related to butyrate-mediated improvements in gut function, nutrient utilization, and immune regulation, as suggested by previous experimental studies [53,54]. These physiological responses may contribute to improved energy allocation and resilience under environmental challenges. Previous studies suggest that butyrate may enhance gut function by modulating the microbiota composition and maintaining epithelial integrity [55,56]. These effects promote rapid growth and strengthen the immune system, making crustaceans more resilient to environmental stressors. In freshwater and marine environments, butyrate supplementation increased the FBW and SR. However, the magnitude of these effects may vary according to environmental conditions and biological characteristics, including water chemistry and microbial communities, which represent potential sources of variability requiring further investigation [57]. These findings highlight the robust benefits of butyrate supplementation across diverse aquatic ecosystems and suggest that specific environmental conditions may influence the mechanisms by which butyrate enhances growth and survival.

The analysis of butyrate application rates provides evidence for its dose-dependent benefits in crustacean aquaculture, particularly for growth performance. Across all environments, butyrate supplementation showed a significant positive effect overall, with doses in the 1–2% range consistently yielding the most pronounced improvements. Optimizing the butyrate dosage is crucial for maximizing the benefits while minimizing costs, as doses exceeding 2% yielded little additional benefit for digestive enzyme activity and growth performance compared to the 1–2% range, whereas feed efficiency continued to improve without a clear plateau. The tendency toward reduced additional benefits at higher inclusion levels is primarily supported by the crustacean data summarized above. As tentative cross-taxa context only, and given that crustacean digestive physiology differs meaningfully from that of finfish, comparable observations have also been reported in largemouth bass (*Micropterus salmoides*) [58]. Similarly, other research reported a plateau in the effects of sodium butyrate on juvenile golden pompano (*Trachinotus ovatus*) at certain dosage levels [32]. These findings highlight the importance of optimizing butyrate supplementation in aquaculture management to improve sustainability and profitability. Careful consideration of dosage can lead to significant improvements in the health and growth performance of aquatic species, reinforcing the need for continued research into the optimal application of butyrate in aquaculture.

The considerable heterogeneity and publication biases observed in the meta-analysis regarding the effects of butyrate supplementation in crustacean aquaculture are consistent with findings from previous studies on similar dietary supplements in aquatic animals. The observed heterogeneity may reflect several factors, including species differences, environmental conditions, experimental protocols, and variation among dietary supplement formulations. For instance, meta-analyses on the impact of probiotics in aquaculture revealed significant variability in outcomes such as FBW, SGR, and SR, attributed to differences in experimental designs and the diversity of organisms tested [59,60]. These studies corroborate the idea that the variability observed in butyrate supplementation studies may result from species-specific factors, environmental conditions, and experimental protocols. Furthermore, Egger’s test indicated significant publication bias across various variables. However, the trim-and-fill method was crucial for evaluating the robustness of this study’s findings and effectively addressing publication bias. This method supported the overall robustness of the observed effects after accounting for potential publication bias, and underscored the importance of statistical methods in ensuring reliable conclusions in scientific studies.

### 4.4. Dose-Dependent Effects of Butyrate on Biological Outcomes

The meta-regression analysis provided valuable insights into the dose-dependent effects of butyrate supplementation on key performance indices in crustacean aquaculture, specifically digestive enzyme activity, feed efficiency, and growth performance (Figure 7, Table 2). The results indicate a significant baseline positive effect of butyrate on digestive enzymes and growth performance, with intercept values of 1.6500 (*p* < 0.0001) and 0.9533 (*p* < 0.0001), respectively. This suggests that butyrate supplementation is generally associated with positive effects on digestive enzyme activity and growth performance across the studies evaluated, although the magnitude of these responses may vary with dose, species, and experimental conditions. These findings align with our earlier discussion, emphasizing the role of butyrate in optimizing nutrient digestion and absorption, which may contribute to the improvements in growth performance observed in the meta-analysis.

However, the meta-regression revealed that the dose–response relationships for digestive enzymes and growth performance were not significant (*p* > 0.05 for both). This contrasts with the findings for feed efficiency, where the slope was significant (Slope = 0.3615, *p* = 0.0101), indicating a dose-dependent response in feed efficiency. This result suggests that increasing the butyrate inclusion may affect feed utilization efficiency, although the contributions of individual indicators (FCR and PER) should be interpreted in light of their biological meaning. The lack of a significant dose–response for digestive enzyme activity and growth performance suggests that while these indices are positively affected by butyrate, their improvement may plateau at certain doses, and further increases in dosage might not yield proportional benefits.

Interestingly, the meta-regression analysis also identified substantial residual heterogeneity for digestive enzymes and growth performance (QE = 209.3381 and QE = 219.6586, *p* < 0.0001), indicating significant variability in these outcomes across studies. This variability may reflect species-specific, environmental, and methodological differences among the included studies, as discussed earlier in Section 4.3. Conversely, feed efficiency did not exhibit significant residual heterogeneity (QE = 56.0930, *p* = 0.2898), suggesting that the improvements may be more consistent across different species and environmental conditions. The significant heterogeneity in digestive enzymes and growth performance underscores the need for further research into the specific environmental and biological factors that may influence butyrate’s efficacy. For example, factors such as salinity, temperature, and microbial composition could interact with butyrate to produce varying effects, as seen in the previous sections.

The findings from this meta-regression analysis reinforce the importance of optimizing butyrate dosages in aquaculture. Although butyrate supplementation benefits crustacean aquaculture systems, the lack of a dose-dependent relationship with digestive enzymes and growth suggests that excessive dosages may not always yield better outcomes. In contrast, the significant dose–response pattern observed for feed efficiency suggests that feed utilization responses may differ from those of digestive enzymes and growth performance; however, further studies are required to determine whether higher inclusion levels provide consistent advantages across species and conditions. These insights further emphasize the need for species-specific and environment-tailored supplementation strategies, which could help mitigate the variability observed across studies and improve the consistency of butyrate’s effects.

Beyond habitat differences, taxonomic variability represents another potential source of heterogeneity. Shrimp, prawns, crayfish, and crabs differ in digestive anatomy, feeding behavior, intestinal microbiota, and metabolic strategies. These biological differences may influence butyrate absorption, microbial interactions, and physiological responses. Therefore, the magnitude of observed effects should be interpreted in light of both environmental and taxonomic contexts.

### 4.5. Potential Mechanisms Underlying Butyrate Effects

**Figure 8:**
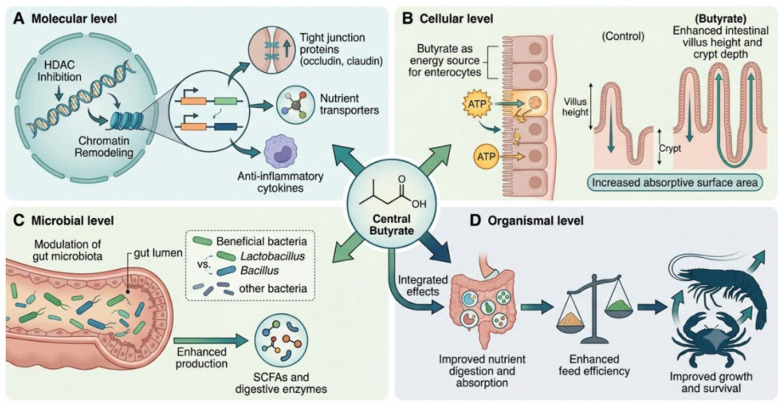
Proposed mechanistic framework for the effects of butyrate on crustacean performance. (**A**) molecular: inhibits HDAC activity, modulating gene expression; (**B**) cellular: serves as an energy source for enterocytes, enhancing intestinal integrity and absorptive surface; (**C**) microbial: modulates gut microbiota, promoting beneficial bacteria that produce SCFAs and digestive enzymes; (**D**) organismal: integrated effects improve feed efficiency, growth, and survival. Arrows indicate the proposed direction of biological interactions, and colors are used for visual distinction among mechanistic levels.

The present meta-analysis provides strong quantitative evidence for the beneficial effects of butyrate supplementation on digestive health, feed efficiency, and growth performance in crustaceans. However, the underlying mechanisms remain incompletely understood. Based on the available literature, several candidate mechanisms can be proposed, and these are summarized conceptually in Figure 8.

First, previous studies suggest that butyrate may enhance intestinal epithelial integrity by promoting the expression of tight junction proteins such as occludin and claudin, which help maintain paracellular permeability and limit pathogen translocation [61,62]. This barrier-enhancing effect has been associated in other animal models with the activation of antioxidant pathways, including Nrf2 signaling, which may reduce oxidative stress in intestinal tissues [63].

Second, butyrate may modulate host immune responses through the regulation of inflammatory pathways, including the inhibition of NF-κB activation and reduction in pro-inflammatory cytokine production (e.g., TNF-α and IL-1β) while potentially promoting anti-inflammatory mediators such as IL-10 [64,65]. This immunomodulatory effect may contribute to the improved survival rates observed in butyrate-supplemented crustaceans, particularly under stress or pathogen challenge conditions.

Third, previous studies indicate that butyrate can influence gut microbial communities by promoting beneficial bacterial groups and modifying the microbial metabolite profile [23,66]. These shifts in microbial community composition may enhance nutrient digestibility and energy harvest, thereby improving the feed efficiency and growth performance.

Importantly, these mechanisms are not mutually exclusive but are likely to operate in a coordinated manner, with potential interactions occurring across molecular, cellular, and organismal levels. Further research, including transcriptomic, metabolomic, and microbiomic studies, is needed to disentangle the relative contributions of these pathways and to identify formulation- and species-specific responses in crustaceans.

### 4.6. Limitations and Future Directions

While this meta-analysis provides quantitative evidence supporting the beneficial effects of butyrate supplementation, several limitations should be acknowledged. First, the substantial heterogeneity observed across studies (I^2^ = 66–97%) indicates that the effects of butyrate vary considerably depending on species, environmental conditions, and experimental protocols. Although we attempted to account for this heterogeneity through subgroup analyses and meta-regression, the limited number of studies per subgroup precluded more detailed analyses. Second, our analysis was based on aggregate data extracted from published studies, which limited our ability to examine individual-level responses or to control for potential confounding factors. Third, the observational nature of the included studies and the absence of direct mechanistic experiments mean that our findings are correlative rather than causal. While we have proposed several plausible mechanisms based on the existing literature, these hypotheses require validation through targeted experimental studies. Fourth, the geographic concentration of studies in Asia (71%) may limit the generalizability of our findings to other aquaculture systems and geographical regions. Future research should therefore include a broader range of species, environmental conditions, and geographic locations to further validate the applicability of butyrate supplementation across global crustacean aquaculture systems. Fifth, the pooled analyses included different butyrate formulations and four taxonomically distinct crustacean groups. Although these sources of variability were recognized and discussed, formulation- and taxon-specific moderator analyses were limited by the number of available studies. Future research should therefore prioritize standardized comparative experiments evaluating different formulations and crustacean taxa under comparable conditions.

## 5. Conclusions

This meta-analysis synthesizing evidence from studies published between 2011 and 2025 indicates that dietary butyrate supplementation is generally associated with improved digestive health, feed utilization, growth performance, and survival in crustaceans. Significant enhancements were observed in digestive enzyme activity, feed efficiency indicators, final body weight, and survival rate across both freshwater and marine production systems. Dose–response analyses suggested that 1–2% inclusion levels generally yielded the most consistent improvements in digestive enzyme activity and growth performance, although feed efficiency responses showed a different pattern, lacking a clear plateau within the evaluated range. Collectively, these mechanisms may contribute to improved nutrient utilization and biological performance involving intestinal barrier function, immune regulation, antioxidant defense, and gut microbiota modulation. Collectively, these mechanisms improve nutrient utilization and biological performance, supporting the use of butyrate as an effective functional feed additive in crustacean aquaculture. Despite the robustness of the overall positive effects, substantial heterogeneity among studies highlights the influence of species-specific and environmental factors. Future research should integrate transcriptomic, metabolomic, and microbiome-based approaches to better elucidate the mechanisms underlying responses to butyrate and to refine species-specific supplementation strategies. Overall, the present study provides quantitative support for the potential use of butyrate as a sustainable nutritional strategy to improve productivity and resilience in crustacean farming.

## Figures and Tables

**Table 1:** Summary of effect sizes and publication bias before and after adjustments.

Variable	k	ES	95–CI	*p*-Value	I^2^ (%)	Egger’s (*p*)	Adj. k	Adj. ES	Adj. 95–CI
ALP (F)	9	2.4008	[0.6091; 4.1925]	0.0086	81.6	0.0002	13	0.4506	[−1.8441; 2.7453]
AMY (F)	4	3.3221	[1.5810; 5.0631]	0.0002	0	0.0002	6	2.8503	[1.3560; 4.3446]
LIP (F)	4	1.1776	[0.1621; 2.1931]	0.023	0	0.0122	6	0.7436	[−0.1296; 1.6168]
TP (F)	4	3.884	[1.8900; 5.8780]	0.0001	0	<0.0001	6	3.2731	[1.5413; 5.0050]
ALP (M)	6	−0.2819	[−1.7242; 1.1603]	0.7016	57.2	0.325	7	0.0863	[−1.4895; 1.6622]
AMY (M)	17	1.3024	[0.2663; 2.3385]	0.0137	67	0.0078	22	0.58	[−0.7927; 1.9527]
LIP (M)	17	1.0809	[0.3654; 1.7964]	0.0031	53.2	<0.0001	23	0.4831	[−0.4136; 1.3798]
TP (M)	7	3.1389	[1.0151; 5.2628]	0.0038	60.2	0.0001	11	1.1141	[−1.2687; 3.4968]
Digestive enzymes		1.8099	[0.9002; 2.7195]	<0.0001	66	0.081	-	1.105	[0.0453; 2.1647]
FCR (F)	4	−0.5528	[−1.3977; 0.2922]	0.1998	0	0.008	4	−0.5528	[−1.3977; 0.2922]
PER (F)	4	1.0319	[0.0855; 1.9784]	0.0326	0	0.0076	5	0.8968	[0.0297; 1.7639]
FCR (M)	6	−1.5647	[−2.5921; −0.5374]	0.0028	37.5	0.0004	7	−1.3456	[−2.3182; −0.3731]
PER (M)	10	0.1633	[−0.3413; 0.6678]	0.5259	0	0.0058	10	0.1633	[−0.3413; 0.6678]
Feed efficiency		−0.2065	[−1.2305; 0.8174]	0.6926	80.5	0.63	-	0.1873	[−0.9484; 1.3229]
FBW (F)	10	1.8956	[0.7136; 3.0775]	0.0017	69.9	<0.0001	14	0.8417	[−1.8952; 3.5786]
SGR (F)	7	0.4616	[−0.0920; 1.0153]	0.1022	0	0.1022	9	0.3658	[−0.1412; 0.8728]
SR (F)	7	3.1756	[1.9304; 4.4207]	<0.0001	19.1	0.0051	10	2.6924	[1.6104; 3.7744]
FBW (M)	38	0.7119	[0.4232; 1.0006]	<0.0001	23.7	<0.0001	51	0.4087	[0.1442; 0.6733]
SGR (M)	3	0.1498	[−0.7782; 1.0778]	0.7517	0	0.0939	3	0.1498	[−0.7782; 1.0778]
SR (M)	28	3.9702	[2.3398; 5.6007]	<0.0001	73.6	<0.0001	39	1.1644	[−1.1838; 3.5126]
Growth Performance		1.9583	[0.6759; 3.2407]	0.0028	97.3	0.2004	-	0.8183	[−0.8446; 2.4813]

Adj. ES, effect size adjusted by the trim-and-fill method; Adj. k, adjusted studies by the trim-and-fill method; Adj. 95–CI, adjusted confidence interval by the trim-and-fill method; ALP, alkaline phosphatase; AMY, amylase; CI, confidence interval (lower and upper); ES, effect size; F, freshwater; FBW, final body weight; FCR, feed conversion ratio; I^2^, percentage variation across studies due to heterogeneity; k, number of studies included; LIP, lipase; M, marine; PER, protein efficiency ratio; SGR, specific growth rate; SR, survival rate; TP, total protease.

**Table 2:** Summary of meta-regression results for biological outcomes.

Outcome	Baseline Effect (Intercept)	Correlation with Application Rate (Slope)	Residual Heterogeneity (I^2^)
Digestive enzymes	1.6500, *p* < 0.0001	0.0287, *p* = 0.8761	209.3381, *p* < 0.0001
Feed efficiency	−0.0186, *p* = 0.9256	0.3615, *p* = 0.0101	56.0930, *p* = 0.2898
Growth performance	0.9533, *p* < 0.0001	−0.0353, *p* = 0.6945	219.6586, *p* < 0.0001

## Data Availability

No new data were created or analyzed in this study. Data sharing is not applicable to this article.

## Supplementary Materials

The following supporting information can be downloaded at: https://www.mdpi.com/article/10.3390/ani16142186/s1, References [67,68,69,70,71,72,73,74,75,76,77] are cited in the Supplementary Materials.
