# Supplementary material for: Effects of Dietary Butyrate Supplementation on Digestive Physiology, Feed Utilization, and Growth Performance in Crustaceans: A Meta-Analysis and Meta-Regression
Source: Animals (Basel). 2026 Jul 14;16(14):2186. doi: 10.3390/ani16142186 (PMC13404980; doi:10.3390/ani16142186)
Supplement: Supplementary file 1 [file animals-16-02186-s001.zip › animals-4426453-Tables and Figures.pdf]

Supplementary data S1

Table S1. Literature included in the meta-analysis. NR = Not Reported.

Study	First Author	Year of Study	Animal Model	Species Identification	Scientific name
**20**	Norha Constanza Bolívar Ramirez	2017	Shrimp	Pacific white shrimp	*Litopenaeus vannamei*
**21**	Nicole Machado Corrêa	2018	Shrimp	Pacific white shrimp	*Litopenaeus vannamei*
**67**	Bruno Corrêa da Silva	2013	Shrimp	Pacific white shrimp	*Litopenaeus vannamei*
**68**	Bruno Corrêa da Silva	2016	Shrimp	Pacific white shrimp	*Litopenaeus vannamei*
**17**	Bruno Corrêa da Silva	2016	Shrimp	Pacific white shrimp	*Litopenaeus vannamei*
**22**	Gaowei Deng	2021	Prawn	Ridgetail white prawn	*Exopalaemon carinicauda*
**23**	Fenglu Han	2020	Crab	Chinese mitten crab	*Eriocheir sinensis*
**18**	Liangfang Liu	2022	Shrimp	Pacific white shrimp	*Litopenaeus vannamei*
**69**	Saori Mine	2011	Shrimp	Pacific white shrimp	*Litopenaeus vannamei*
**70**	Mpwaga Alatwinusa Yohana	2025	Shrimp	Pacific white shrimp	*Litopenaeus vannamei*
**71**	Nischal	2025	Shrimp	Pacific white shrimp	*Litopenaeus vannamei*
**72**	Oliveira Maria Érica da Silva	2025	Shrimp	Pacific white shrimp	*Litopenaeus vannamei*
**15**	Hongyu Peng	2025	Crab	Mud crab	*Scylla paramamosain*
**49**	Artur N. Rombenso	2020	Shrimp	Black tiger shrimp	*Penaeus monodon*
**16**	Omid Safari	2021	Crayfish	Narrow clawed crayfish	*Astacus leptodactylus leptodactylus*
**73**	Bruno Corrêa Silva	2016	Shrimp	Pacific white shrimp	*Litopenaeus vannamei*
**29**	Bruno Corrêa Silva	2016	Shrimp	Pacific white shrimp	*Litopenaeus vannamei*
**74**	Suhaila Abdul Sukor	2016	Crab	Blue swimmer crab	*Portunus pelagicus*
**19**	Peyman Yarahmadi	2022	Shrimp	Pacific white shrimp	*Litopenaeus vannamei*
**66**	Minghan Yin	2025	Shrimp	Pacific white shrimp	*Litopenaeus vannamei*
**75**	Mpwaga Alatwinusa Yohana	2024	Shrimp	Pacific white shrimp	*Litopenaeus vannamei*
**76**	Mpwaga Alatwinusa Yohana	2024	Shrimp	Pacific white shrimp	*Litopenaeus vannamei*

**77**	Chongyang Xiao	2021	Crayfish	Red swamp crayfish	*Procambarus clarkii*

Table S2. Literature included in the meta-analysis. NR = Not Reported.

Study	Developmental Stage	Habitat Type	Stocking Density	Experimental Replicates
**20**	Juvenile	Marine	40 individuals/m³ (30 shrimp in 800 L tanks)	4
**21**	Post-larvae	Marine	2,250 individuals/m³	3
**67**	Juvenile	Marine	NR	3
**68**	Juvenile	Marine	250 individuals/m³ (200 shrimps in 800 L tanks)	4
**17**	Juvenile	Marine	12 individuals/m² (150 shrimps in 6000 L tanks)	3
**22**	Juvenile	Marine	40 individuals/m³ (40 shrimps in 100 L tanks)	3
**23**	Juvenile	Freshwater	35 crabs per tank	5
**18**	Juvenile	Marine	30 individuals/m³	3
**69**	Juvenile	Marine	NR	3
**70**	Juvenile	Marine	40 shrimp/tank	3
**71**	Post-larvae	Marine	NR	NR
**72**	Post-larvae to grow-out phase	Marine	160 shrimp/replicate; Grow-out: 75 shrimp/replicate	4
**15**	Juvenile	Marine	12 individuals/m³	3
**49**	Juvenile	Marine	125 individuals/m³	4
**16**	Juvenile	Freshwater	18 individuals/m³	3
**73**	Juvenile	Marine	NR	4
**29**	Juvenile	Marine	150 individuals/m³	3
**74**	Early juveniles	Marine	NR	4
**19**	Juvenile	Marine	NR	3
**66**	Juvenile	Marine	30 shrimp/tank	3
**75**	Juvenile	Marine	NR	3
**76**	Juvenile	Marine	10 individuals/m³	3
**77**	Juvenile	Freshwater	NR	3

Table S3. Literature included in the meta-analysis. NR = Not Reported.

Study	Initial Body Weight	Acclimation Period	Water Temperature	pH Levels
**20**	5.28 g	NR	29.56 ± 0.30°C	8.37 ± 0.08
**21**	0.03 g	NR	28.0 ± 1.0°C	8.30 ± 0.12
**67**	9.6 g	NR	26-29°C	6.2 (diet pH), 7.1 (intestinal pH)
**68**	3.96 g	NR	29.1 ± 0.5°C	7.54 ± 0.13
**17**	2.53 g	NR	27.5-29.4°C	7.9 (7.6-8.1)
**22**	0.0146 g	NR	26-30°C	7.8-8.1
**23**	0.33 ± 0.01 g	14 days	25-28°C	7.6-8.4
**18**	0.52 ± 0.03 g	NR	28-30°C	7.8-8.5
**69**	NS	NR	NR	NR
**70**	0.19 g	3 weeks	28-30°C	7.5–8.0
**71**	NR	NR	NR	NR
**72**	93.18 ± 1.617 mg	19 days	29.18–29.85 °C	8.01–8.24
**15**	10.56 g	7 days	28.72 ± 0.06°C	7.54 ± 0.02
**49**	0.72 ± 0.01 g	NR	28.8 ± 0.8°C	NR
**16**	4.38 ± 0.08 g	NR	25.3°C	7.31 ± 0.67
**73**	3.96 ± 0.04 g	NR	29 ± 0.5°C	NR
**29**	0.49 ± 0.02 g	NR	27.2 ± 0.2°C	8.08 ± 0.01
**74**	5.9 mg	NR	26-28°C	NS
**19**	4.89 ± 0.32 g	NR	28°C	NS
**66**	0.19 g	14 days	28-30°C	7.5–8.0
**75**	12.76 ± 0.38 g	NR	28-30°C	NR
**76**	0.18 g	28 days	26.0 - 31.5°C (normal); 38°C (heat stress)	7.9 - 8.1
**77**	16 ± 1.80 g	7 days	25°C	NR

Table S4. Literature included in the meta-analysis. NR = Not Reported.

Study	Dissolved Oxygen	Ammonia Concentration	Nitrite Concentration	Nitrate Concentration
**20**	6.07 ± 0.05 mg/L	0.55 ± 0.19 mg/L	0.13 ± 0.11 mg/L	NR
**21**	5.0 ± 1.0 mg/L	1.0 ± 0.03 mg/L	0.38 ± 0.65 mg/L	13.35 ± 2.57 mg/L
**67**	NR	0.91-0.35 mg/L	0.00-0.03 mg/L	NS
**68**	5.3 ± 0.1 mg/L	0.18 ± 0.11 mg/L	0.57 ± 0.14 mg/L	55.2 ± 16.1 mg/L
**17**	4.9 mg/L (4.4-5.8 mg/L)	0.6 mg/L (0.1-1.4 mg/L)	0.1 mg/L (0.0-0.3 mg/L)	NS
**22**	NS	<0.05 mg/L	NR	NR
**23**	7.0 mg/L	NR	NR	NR
**18**	5.1-6.7 mg/L	<0.1 mg/L	NR	NR
**69**	NR	NR	NR	NR
**70**	6.5 mg/L	NR	NR	NR
**71**	NR	NR	NR	NR
**72**	6.73–7.35 mg/L	0–0.025 mg/L	0.02–0.04 mg/L	NR
**15**	7.71 ± 0.02 mg/L	NR	NR	NR
**49**	5.8 ± 0.6 mg/L	NR	NR	NR
**16**	6.26 ± 0.78 mg/L	<0.06 mg/L	<0.6 mg/L	NR
**73**	NR	NR	NR	NR
**29**	5.14 ± 0.01 mg/L	NR	NR	NR
**74**	NR	NR	NR	NR
**19**	NR	NR	NR	NR
**66**	6.5 mg/L	0.2 mg/L	0.01 mg/L	NS
**75**	NR	NR	NR	NR
**76**	5.6 - 6.5 mg/L	NR	NR	NR
**77**	NR	NR	NR	NR

Table S5. Literature included in the meta-analysis. NR = Not Reported.

Study	Salinity Levels	Dietary Type
**20**	30 ppt	Diets supplemented with butyrate, probiotic, butyrate + probiotic, and control (no additives)
**21**	31.80 ± 1.45 ppt	Dietary supplementation with probiotic (Lactobacillus plantarum) and sodium butyrate (2.0%)
**67**	33-35 ppt	Diets supplemented with 2% of different salts of organic acids (sodium acetate, sodium butyrate, sodium citrate, sodium formate, sodium lactate, and sodium propionate)
**68**	35.3 ± 1.5 ppt	Diets supplemented with 2% sodium butyrate and 2% polyhydroxybutyrate (PHB)
**17**	35 ppt (34-37 ppt)	Diets supplemented with sodium propionate and sodium butyrate at concentrations of 0.5%, 1%, and 2%
**22**	24.5-26.5 ppt	Diets supplemented with 0.25%, 0.5%, and 1% of sodium acetate, sodium propionate, and sodium butyrate
**23**	NR	Dietary supplementation with sodium butyrate (SBT)
**18**	NR	Dietary supplementation with sodium butyrate
**69**	NR	Dietary supplementation with organic acids (formic acid, acetic acid, propionic acid, and butyric acid)
**70**	32 ppt	Distillers Dried Grains with Solubles (DDGS) supplemented with butyric acid (BA)
**71**	NR	Sodium butyrate (1%, 2%, 3%)
**72**	NR	Basal shrimp diet supplemented with protected sodium butyrate
**15**	24.32 ± 0.09 ppt	Dietary supplementation with sodium butyrate (NaB)
**49**	NR	Dietary supplementation with butyrate (BUT), succinate (SUC), and fumarate (FUM) individually or in combination (ALL)
**16**	NR	Dietary supplementation with encapsulated organic salts (Na-acetate, Na-butyrate, Na-lactate, Na-propionate)
**73**	NR	Dietary supplementation with sodium butyrate and polyhydroxybutyrate (PHB)
**29**	38.93 ppt	Butyrate
**74**	32 ppt	Dietary supplementation with organic acid salts (sodium acetate, sodium citrate, sodium butyrate, or sodium propionate at 2%)
**19**	NR	Dietary supplementation with sodium butyrate
**66**	32 ppt	Butyric acid supplementation (in vitro and in vivo assays)
**75**	NR	Dietary supplementation with butyric acid (BA)
**76**	32 - 35 ppt	Dietary supplementation with butyric acid (BA)
**77**	NR	Dietary supplementation with sodium butyrate (SB)

Table S6. Literature included in the meta-analysis. NR = Not Reported.

Study	Duration of Experiment	Feeding Schedule	Pathogen Challenge
**20**	28 days (4 weeks)	08:00, 12:00, 14:00, and 17:00 h	*Vibrio alginolyticus*
**21**	35 days	08:00, 11:00, 14:00, 17:00	NR
**67**	14 days (intestinal bacterial count alteration), 14 days (feed intake), 35 days (digestibility assays)	09:00 and 14:00 h (for digestibility assays), NS (for other assays)	*Vibrio harveyi, V. alginolyticus, and V. anguillarum*
**68**	42 days	08:00, 11:00, 14:00, and 17:00 h	NR
**17**	47 days	08:00, 11:00, 14:00, and 17:00 h	NR
**22**	56 days	08:00, 14:00, and 20:00 h	*Vibrio parahaemolyticus*
**23**	56 days	NR	NR
**18**	NR	NR	NR
**69**	NR	NR	White spot syndrome virus (WSSV)
**70**	56 days	4 times/day	NR
**71**			
**72**			
**15**	63 days	08:30 and 17:30	NR
**49**	42 days	11:00 (manual), 18:00, 21:00, 00:00, 03:00 (automatic)	NR
**16**	63 days	08:00, 14:00, 20:00	NR
**73**	42 days	08:00, 11:00, 14:00, 17:00	NR
**29**	45 days	NR	NR
**74**	20 days	Twice daily	NR
**19**	60 days	09:00, 11:00, 14:00, 17:00, 20:00, 23:00	White spot syndrome virus (WSSV)
**66**	56 days	2 times/day	Vibrio parahaemolyticus challenge
**75**	8 weeks (feeding period) + 1 hour (cold stress challenge)	NR	Cold stress challenge
**76**	56 days	4 times/day	NR
**77**	NR	NR	White spot syndrome virus (WSSV)

Supplementary data S2

A. Digestive enzymes

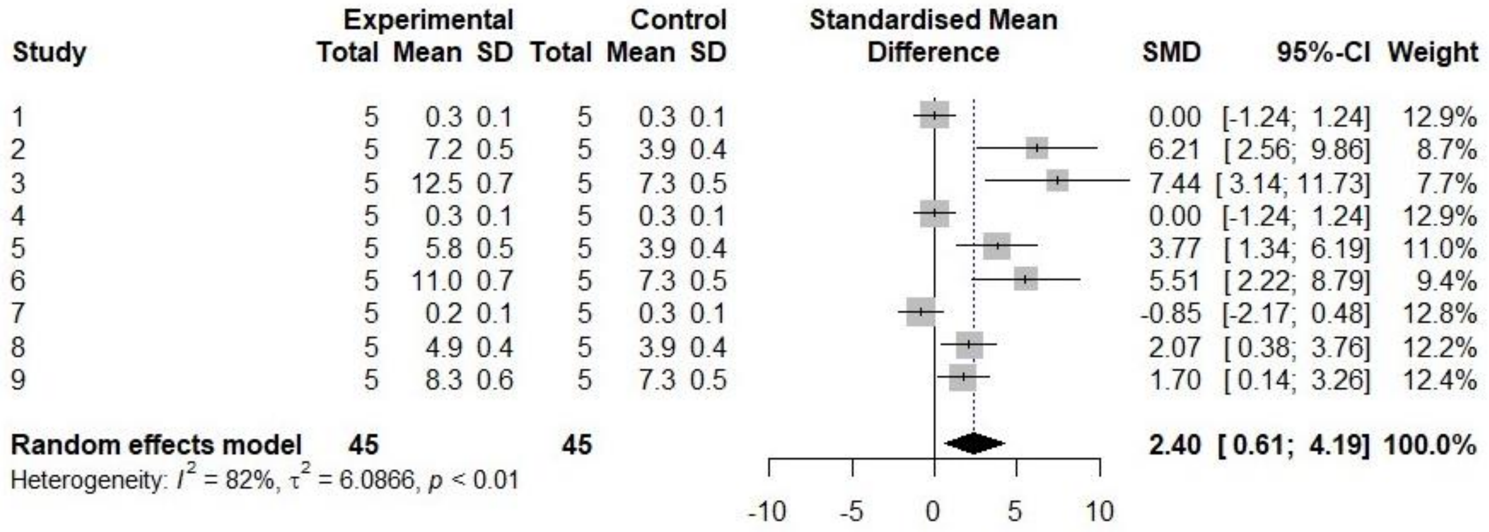

Figure S1. Effect size of alkaline phosphatase in freshwater species supplemented with butyrate.

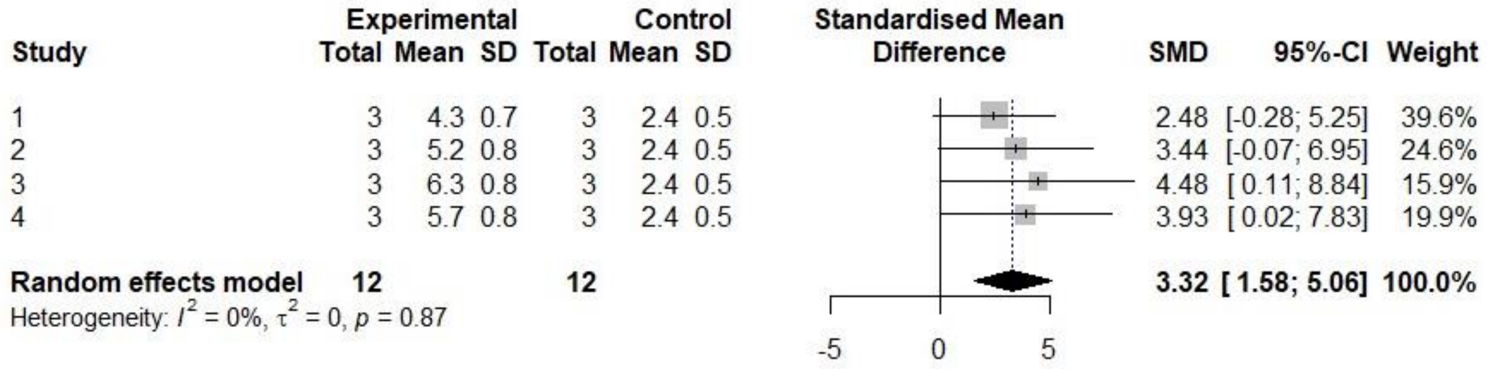

Figure S2. Effect size of amylase in freshwater species supplemented with butyrate.

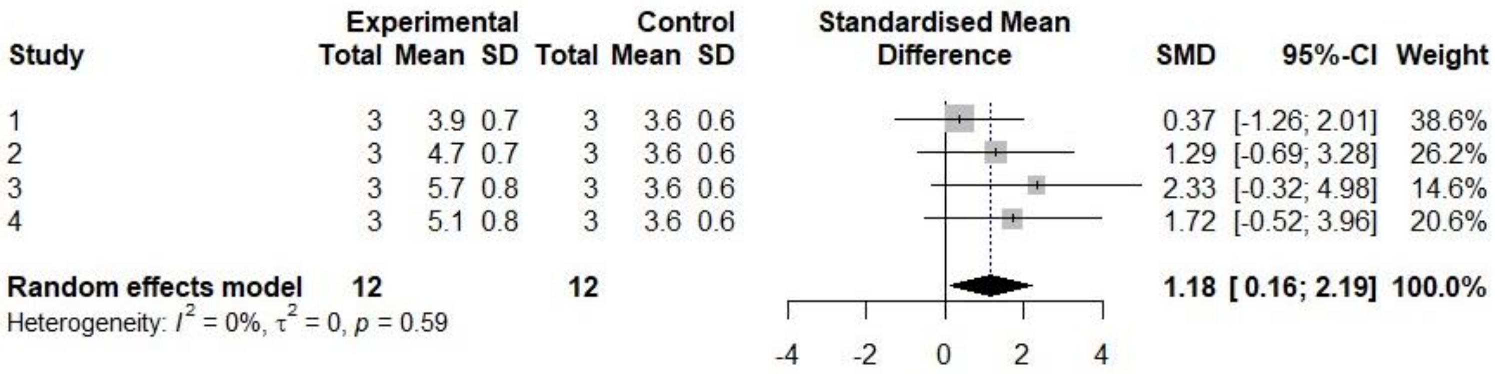

Figure S3. Effect size of lipase in freshwater species supplemented with butyrate.

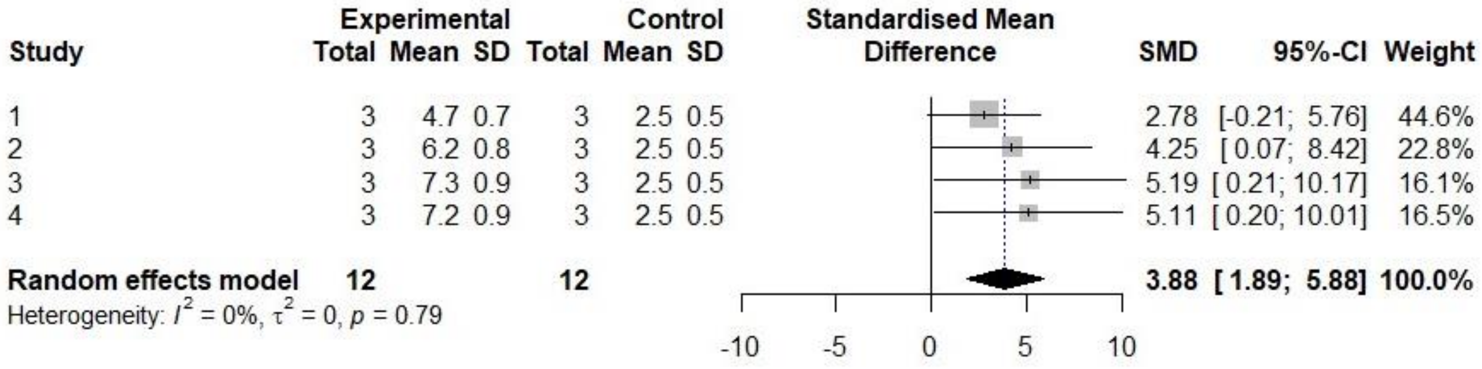

Figure S4. Effect size of total protease in freshwater species supplemented with butyrate.

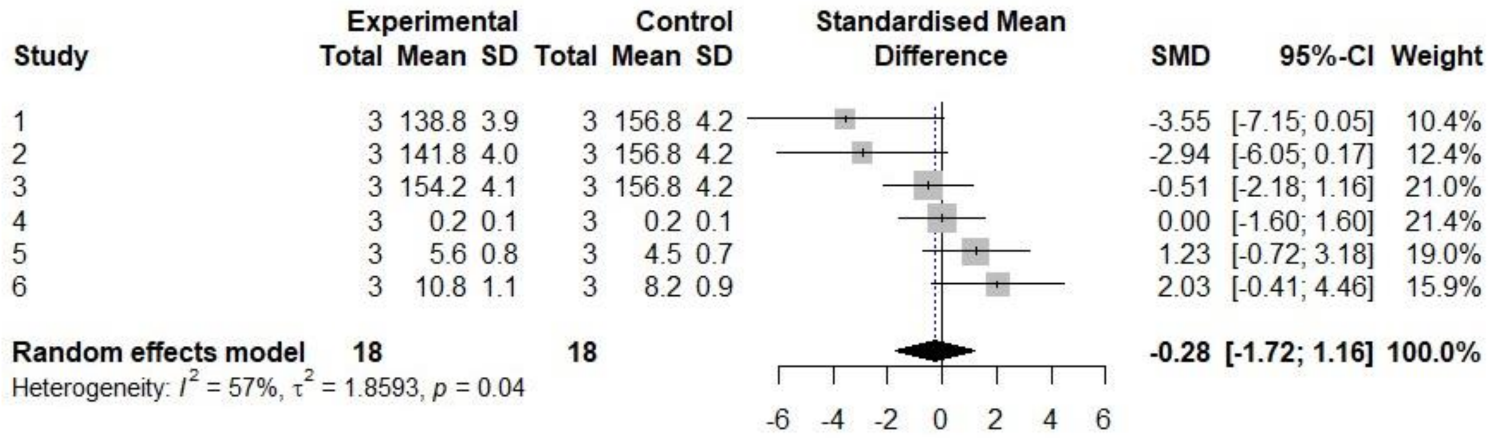

Figure S5. Effect size of alkaline phosphatase in marine species supplemented with butyrate.

Study	Experimental Total	Experimental Mean	Experimental SD	Control Total	Control Mean	Control SD	SMD	95%-CI	Weight
1	3	15.3	1.3	3	15.6	1.3	-0.17	[-1.78; 1.44]	9.1%
2	3	15.4	1.3	3	15.6	1.3	-0.12	[-1.72; 1.49]	9.1%
3	3	16.0	1.3	3	15.6	1.3	0.25	[-1.36; 1.87]	9.0%
4	3	0.6	0.3	3	0.6	0.3	0.00	[-1.60; 1.60]	9.1%
5	3	83.1	3.0	3	73.8	2.9	2.50	[-0.28; 5.27]	6.3%
6	3	151.4	4.1	3	138.5	3.9	2.57	[-0.26; 5.40]	6.2%
7	4	4.7	0.5	4	3.9	0.5	1.38	[-0.28; 3.05]	8.9%
8	4	17.9	1.1	4	31.6	1.4	-9.53	[-16.06; -3.01]	2.1%
9	4	15745.3	31.4	4	12624.2	28.1	91.05	[30.14; 151.96]	0.0%
10	4	1757.8	10.5	4	1660.9	10.2	8.14	[2.52; 13.76]	2.6%
11	3	40.0	2.1	3	38.3	2.1	0.64	[-1.06; 2.34]	8.8%
12	3	46.7	2.3	3	38.3	2.1	3.06	[-0.15; 6.27]	5.5%
13	3	50.7	2.4	3	38.3	2.1	4.43	[0.10; 8.77]	3.8%
14	3	50.7	2.4	3	38.3	2.1	4.43	[0.10; 8.77]	3.8%
15	3	51.0	2.4	3	38.3	2.1	4.54	[0.12; 8.97]	3.7%
16	3	55.0	2.5	3	40.7	2.1	4.96	[0.18; 9.73]	3.3%
17	3	38.3	2.1	3	40.7	2.1	-0.89	[-2.68; 0.90]	8.6%
**Random effects model**	**55**			**55**			**1.30**	**[0.27; 2.34]**	**100.0%**

Heterogeneity: $I^2 = 67\%$, $\tau^2 = 2.4145$, $p < 0.01$

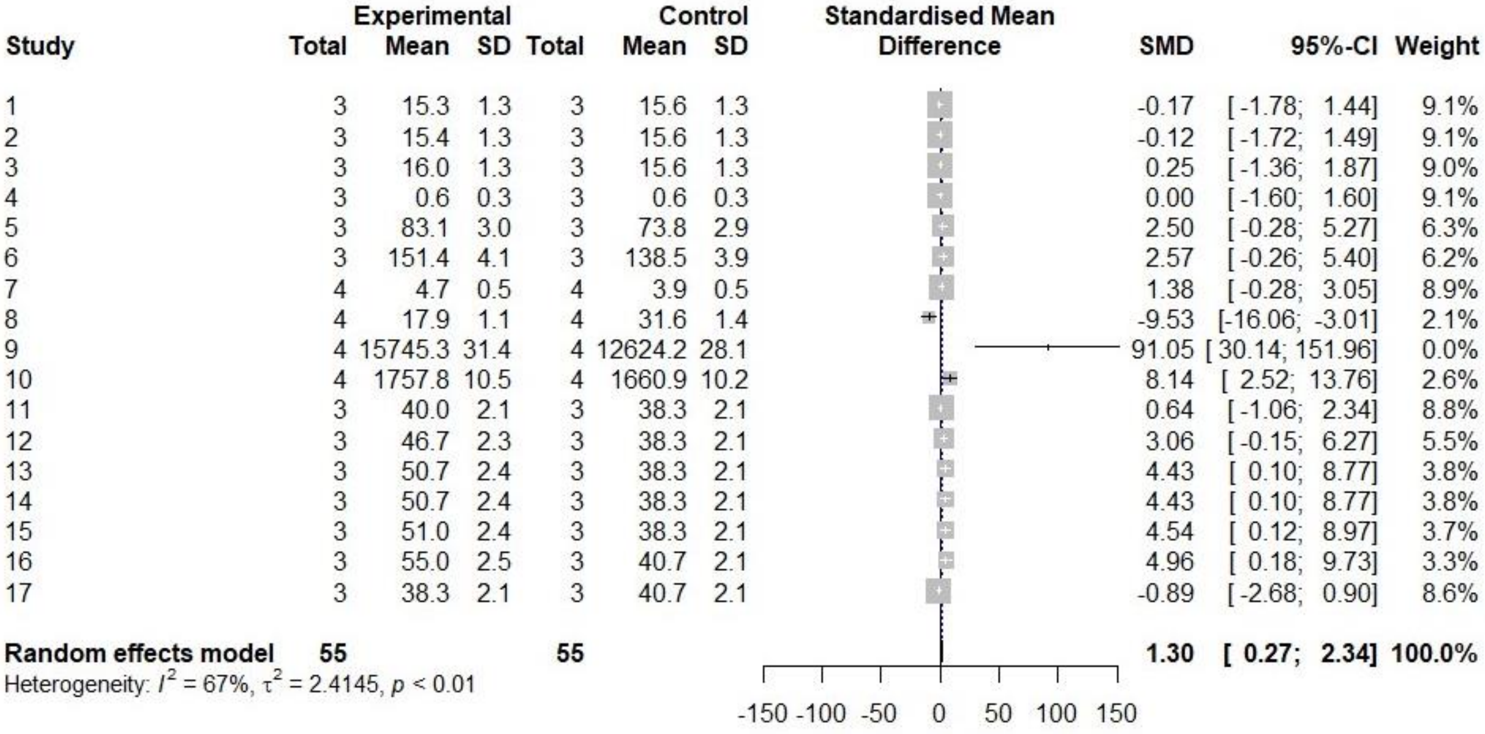

Figure S6. Effect size of amylase in marine species supplemented with butyrate.

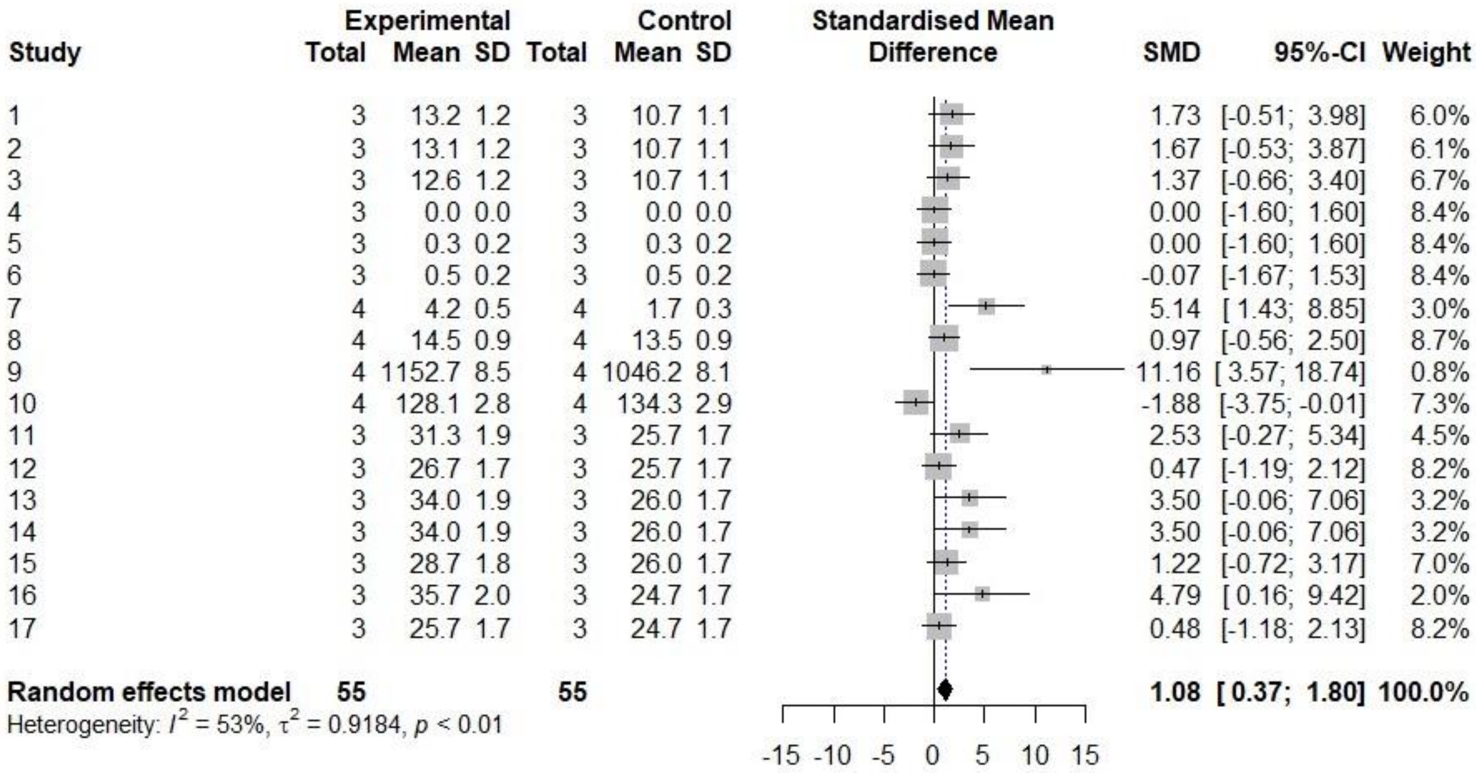

Figure S7. Effect size of lipase in marine species supplemented with butyrate.

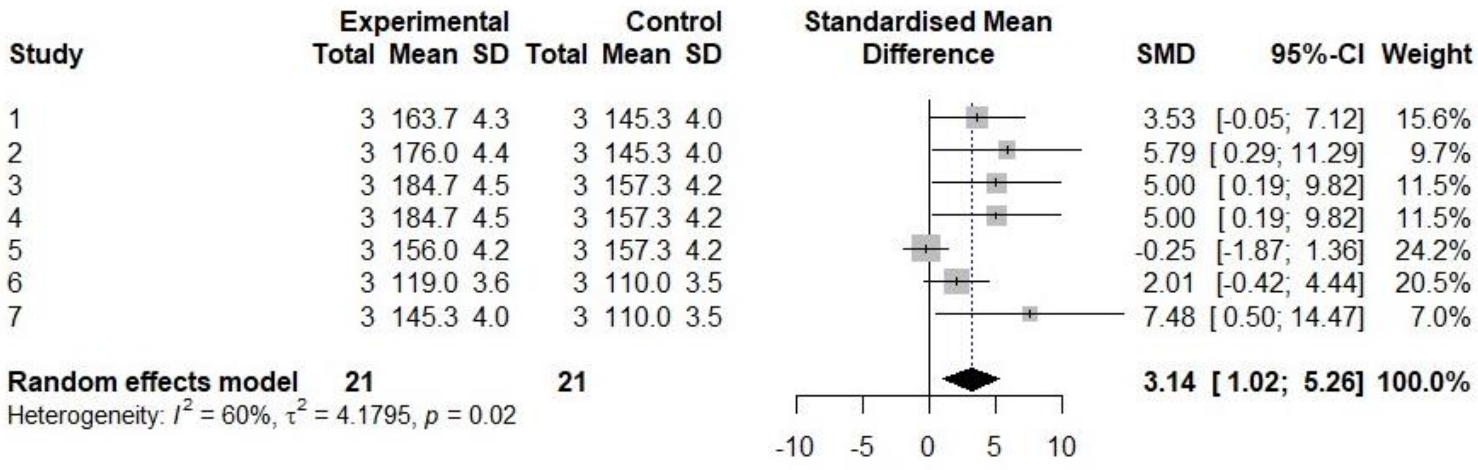

Figure S8. Effect size of total protease in marine species supplemented with butyrate.

B. Feed efficiency

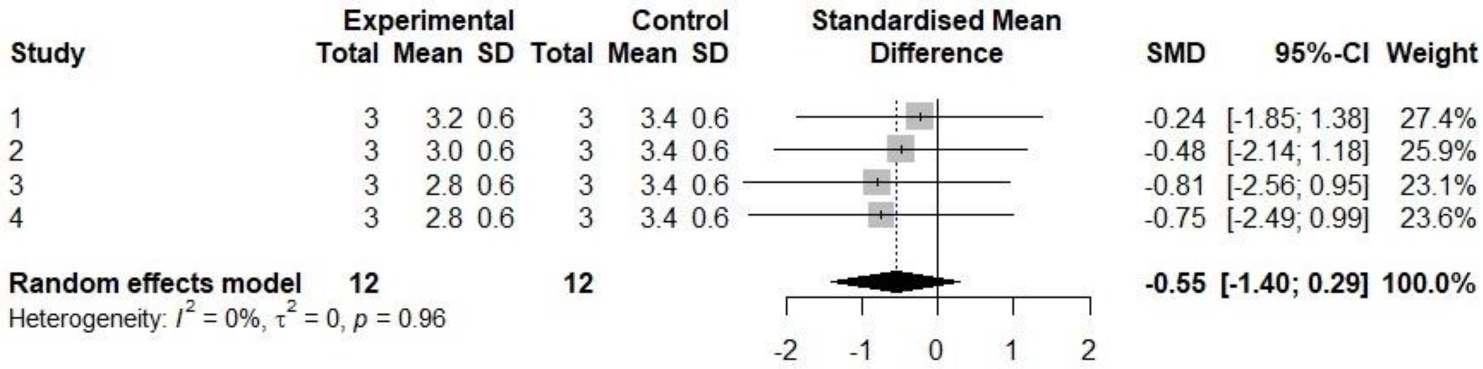

Figure S9. Effect size of feed conversion ratio in freshwater species supplemented with butyrate.

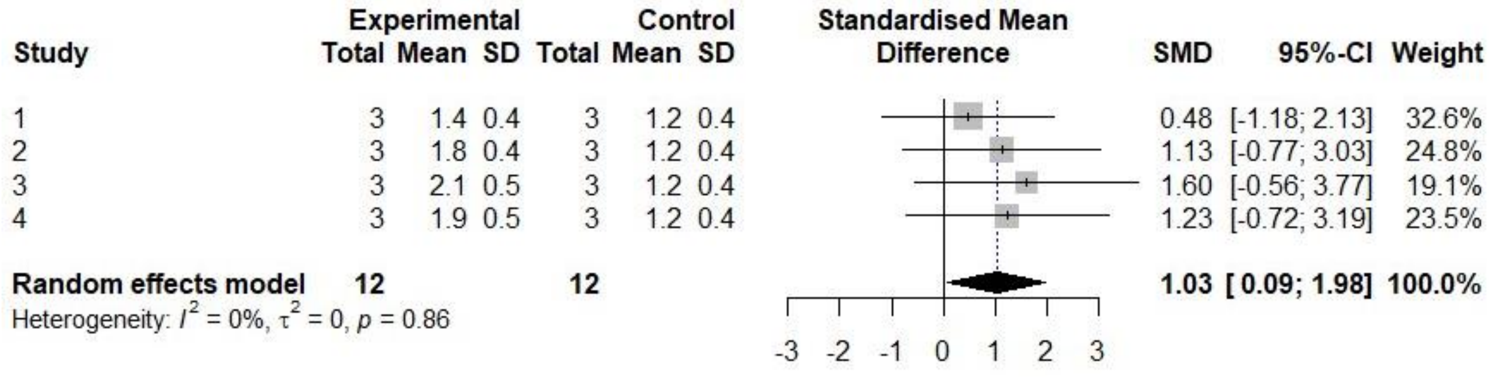

Figure S10. Effect size of protein efficiency ratio in freshwater species supplemented with butyrate.

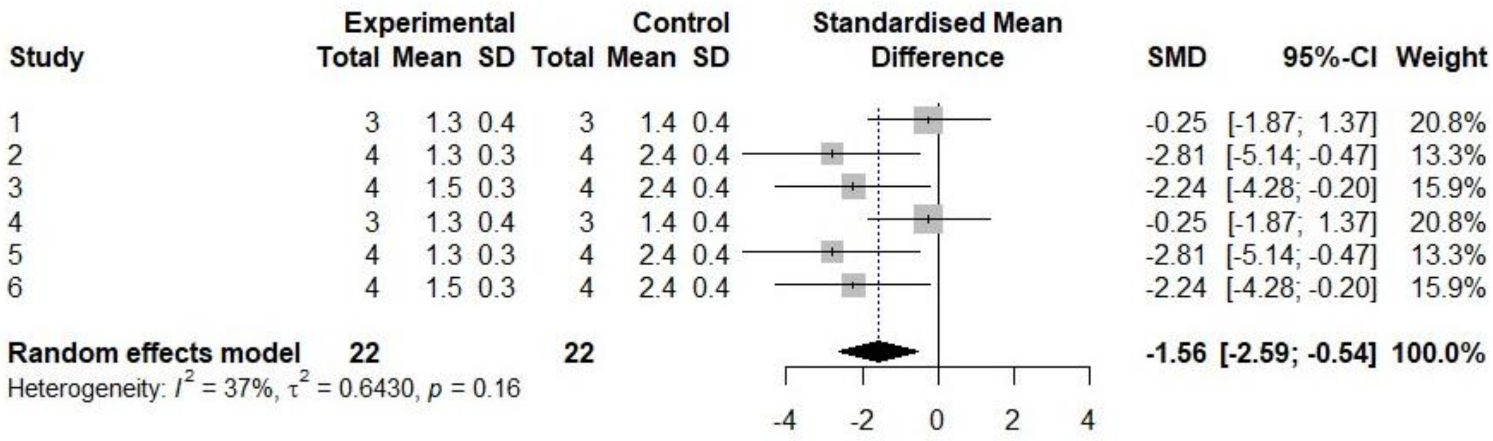

Figure S11. Effect size of feed conversion ratio in marine species supplemented with butyrate.

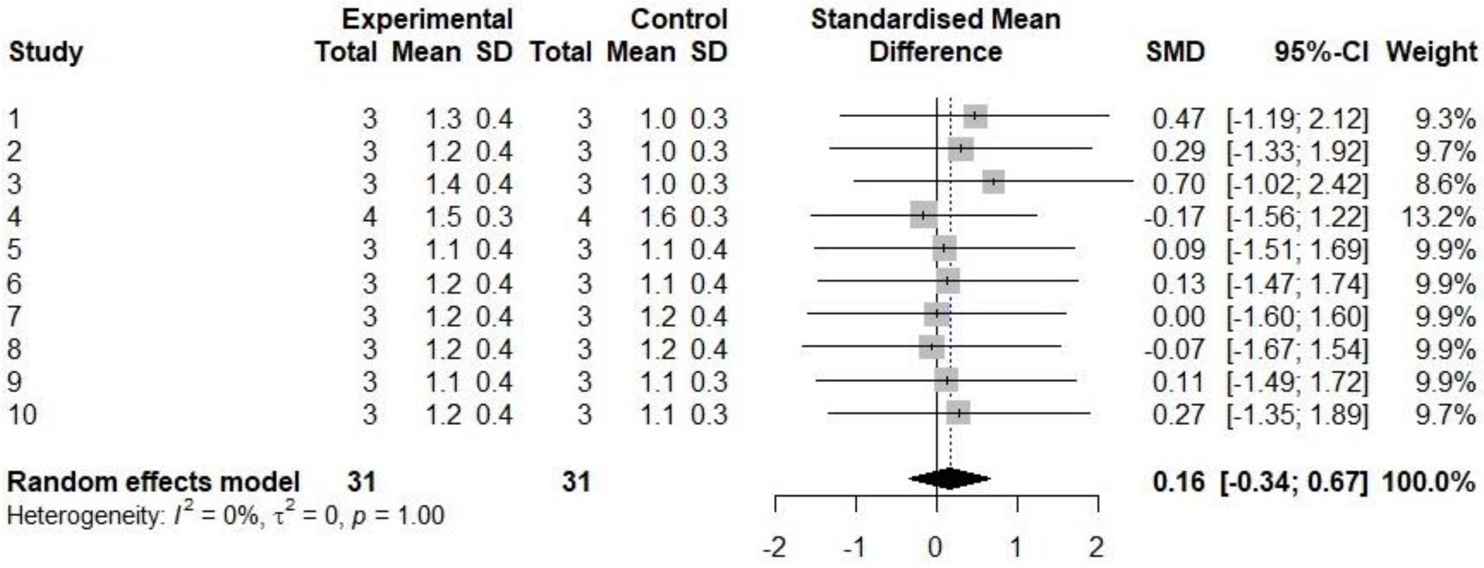

Figure S12. Effect size of protein efficiency ratio in marine species supplemented with butyrate.

C. Growth performance

Study	Experimental Total	Experimental Mean	Experimental SD	Control Total	Control Mean	Control SD	SMD	95%-CI	Weight
1	3	10.5	1.1	3	9.8	1.0	0.52	[-1.15; 2.19]	12.8%
2	3	11.7	1.1	3	9.8	1.0	1.38	[-0.65; 3.42]	11.4%
3	3	13.5	1.2	3	9.8	1.0	2.56	[-0.26; 5.38]	8.7%
4	3	12.5	1.2	3	9.8	1.0	1.94	[-0.44; 4.31]	10.2%
5	5	2.2	0.3	5	1.9	0.3	0.84	[-0.48; 2.16]	14.2%
6	5	2.1	0.3	5	1.9	0.3	0.70	[-0.60; 1.99]	14.3%
7	5	2.0	0.3	5	1.9	0.3	0.29	[-0.96; 1.54]	14.5%
8	5	555.8	4.7	5	484.4	4.4	14.13	[6.22; 22.04]	2.0%
9	5	542.8	4.7	5	484.4	4.4	11.64	[5.08; 18.19]	2.7%
10	5	505.4	4.5	5	484.4	4.4	4.26	[1.60; 6.93]	9.2%
**Random effects model**	**42**			**42**			**1.90**	**[0.71; 3.08]**	**100.0%**

Heterogeneity: $I^2 = 70\%$, $\tau^2 = 2.1063$, $p < 0.01$

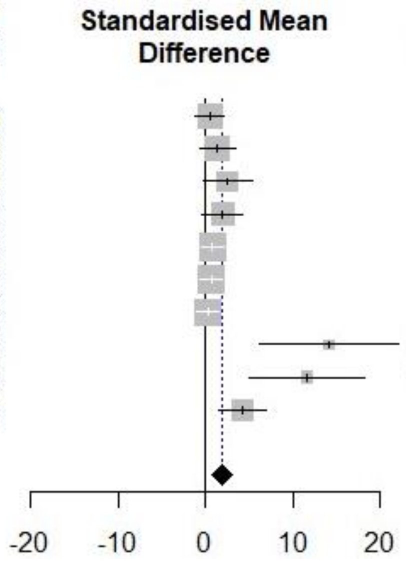

Figure S13. Effect size of final body weight in freshwater species supplemented with butyrate.

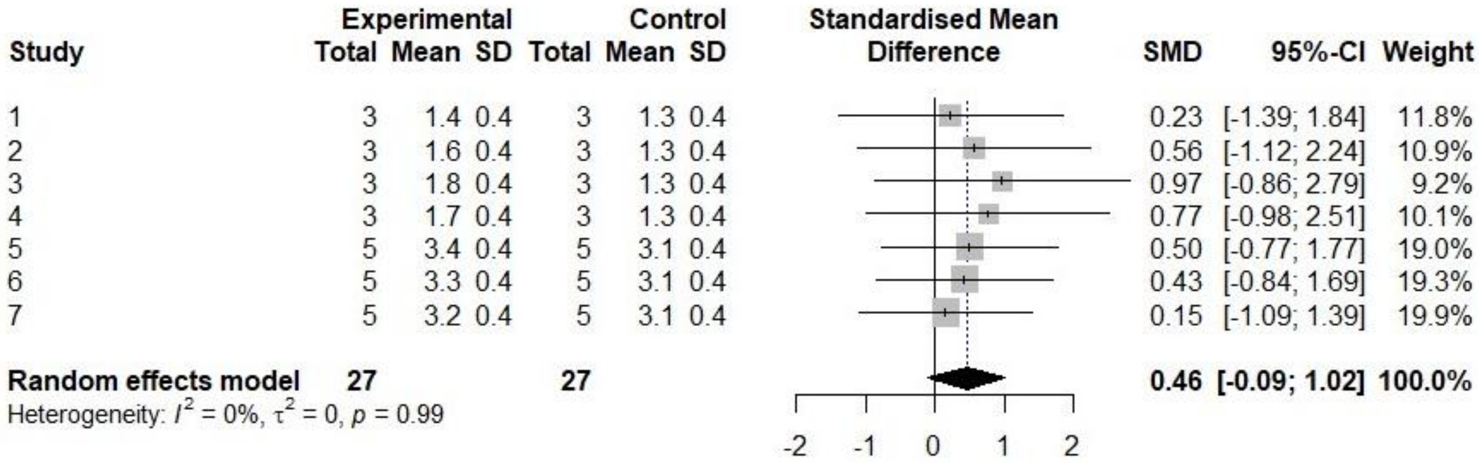

Figure S14. Effect size of specific growth rate in freshwater species supplemented with butyrate.

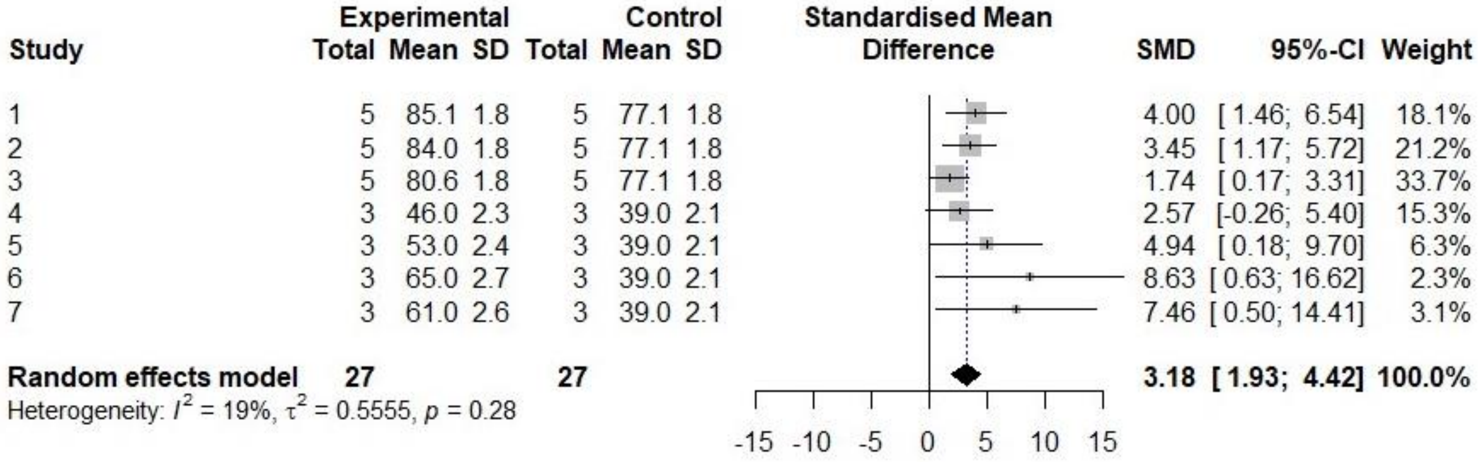

Figure S15. Effect size of survival rate in freshwater species supplemented with butyrate.

Study	Experimental Total	Experimental Mean	Experimental SD	Control Total	Control Mean	Control SD	SMD	95%-CI	Weight
1	4	12.1	0.9	4	12.6	0.9	-0.46	[-1.88; 0.96]	4.1%
2	4	1.7	0.3	4	1.8	0.3	-0.26	[-1.66; 1.14]	4.3%
3	4	12.3	0.9	4	12.6	0.9	-0.23	[-1.62; 1.17]	4.3%
4	4	1.8	0.3	4	1.8	0.3	-0.18	[-1.57; 1.21]	4.3%
5	3	10.6	1.1	3	9.3	1.0	0.97	[-0.86; 2.79]	2.5%
6	3	1.2	0.4	3	0.9	0.3	0.60	[-1.09; 2.29]	2.9%
7	3	10.2	1.1	3	9.3	1.0	0.71	[-1.02; 2.44]	2.8%
8	3	1.1	0.4	3	0.9	0.3	0.49	[-1.17; 2.15]	3.0%
9	3	10.8	1.1	3	9.3	1.0	1.13	[-0.77; 3.03]	2.3%
10	3	1.2	0.4	3	0.9	0.3	0.69	[-1.03; 2.41]	2.8%
11	4	13.3	0.9	4	13.3	0.9	0.01	[-1.38; 1.40]	4.3%
12	4	1.6	0.3	4	1.6	0.3	0.03	[-1.36; 1.41]	4.3%
13	3	413.2	6.8	3	401.9	6.7	1.34	[-0.67; 3.36]	2.1%
14	3	447.3	7.0	3	401.9	6.7	5.28	[0.23; 10.33]	0.3%
15	3	433.2	6.9	3	401.9	6.7	3.68	[-0.03; 7.38]	0.6%
16	3	8.9	1.0	3	7.6	0.9	1.07	[-0.80; 2.94]	2.4%
17	4	25.8	1.3	4	19.3	1.1	4.75	[1.29; 8.22]	0.7%
18	4	28.3	1.3	4	11.6	0.8	13.00	[4.20; 21.80]	0.1%
19	4	28.4	1.3	4	19.3	1.1	6.48	[1.93; 11.02]	0.4%
20	4	28.3	1.3	4	11.6	0.8	13.00	[4.20; 21.80]	0.1%
21	4	3.6	0.5	4	2.9	0.4	1.35	[-0.30; 3.00]	3.0%
22	4	3.9	0.5	4	2.9	0.4	1.88	[0.01; 3.76]	2.4%
23	4	0.5	0.2	4	0.4	0.2	0.51	[-0.92; 1.94]	4.1%
24	4	2.9	0.4	4	2.2	0.4	1.52	[-0.20; 3.23]	2.8%
25	4	0.5	0.2	4	0.4	0.2	0.51	[-0.92; 1.94]	4.1%
26	4	3.2	0.4	4	2.2	0.4	2.11	[0.13; 4.09]	2.1%
27	3	18.3	1.4	3	17.3	1.4	0.57	[-1.11; 2.25]	3.0%
28	3	13.5	1.2	3	12.5	1.2	0.66	[-1.05; 2.38]	2.8%
29	3	19.2	1.5	3	17.3	1.4	1.03	[-0.82; 2.88]	2.4%
30	3	14.2	1.2	3	12.5	1.2	1.12	[-0.78; 3.01]	2.3%
31	3	18.9	1.4	3	17.6	1.4	0.71	[-1.01; 2.44]	2.8%
32	3	14.0	1.2	3	13.1	1.2	0.58	[-1.10; 2.27]	2.9%
33	3	18.7	1.4	3	17.6	1.4	0.63	[-1.07; 2.34]	2.9%
34	3	13.7	1.2	3	13.1	1.2	0.39	[-1.25; 2.03]	3.1%
35	3	16.9	1.4	3	15.9	1.3	0.57	[-1.11; 2.26]	2.9%
36	3	11.8	1.1	3	10.9	1.1	0.64	[-1.06; 2.34]	2.9%
37	3	17.6	1.4	3	15.9	1.3	0.98	[-0.85; 2.81]	2.5%
38	3	12.8	1.2	3	10.9	1.1	1.28	[-0.70; 3.26]	2.1%
**Random effects model**	**130**			**130**			**0.71**	**[0.42; 1.00]**	**100.0%**

Heterogeneity: $I^2 = 24\%$, $\tau^2 < 0.0001$, $p = 0.10$

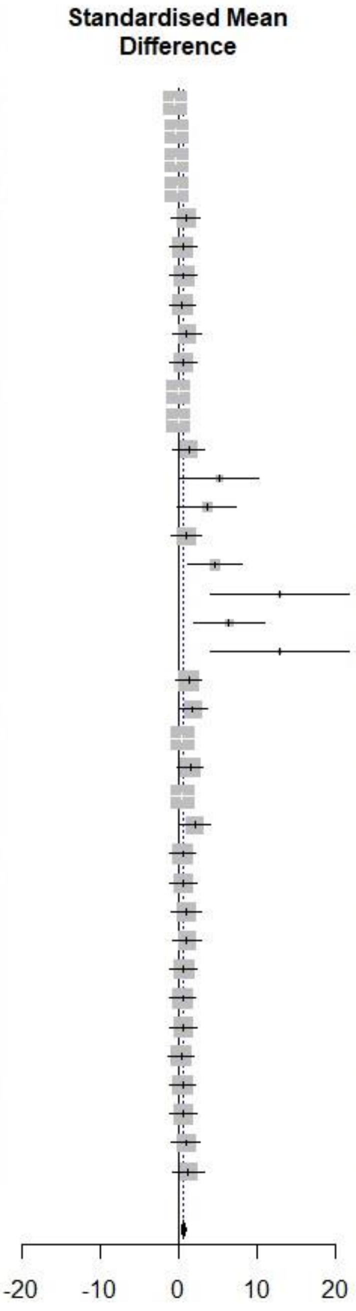

Figure S16. Effect size of final body weight in marine species supplemented with butyrate.

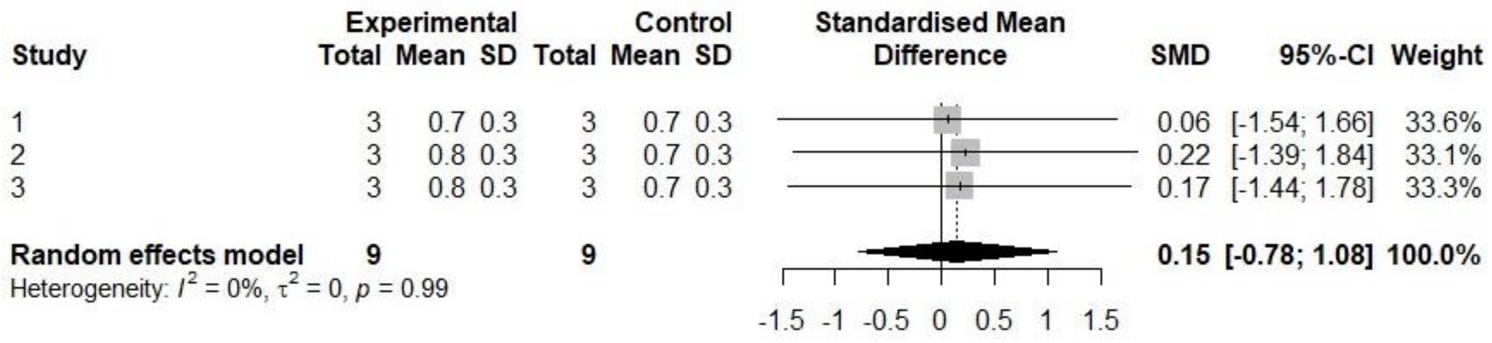

Figure S17. Effect size of specific growth rate in marine species supplemented with butyrate.

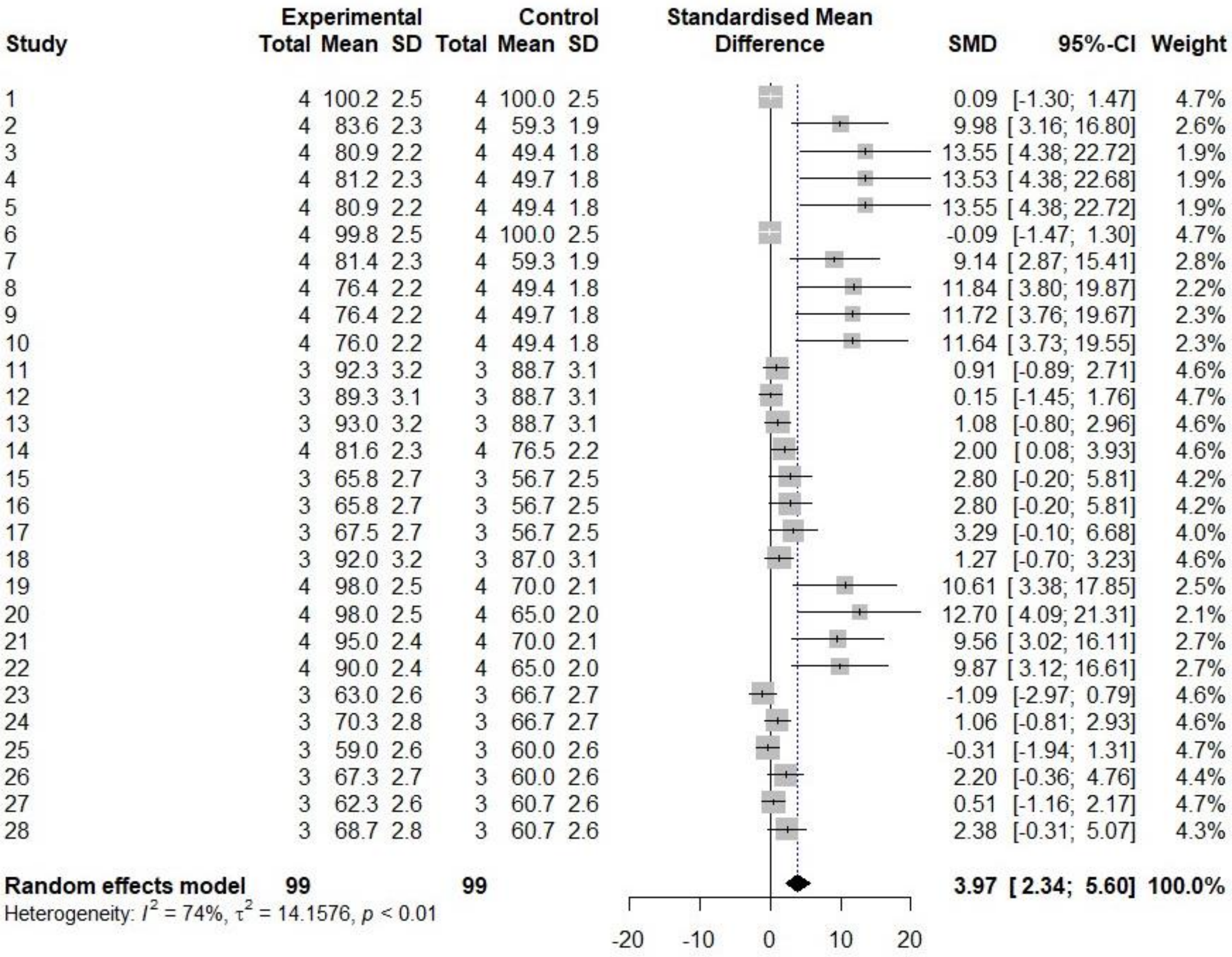

Figure S18. Effect size of survival rate in marine species supplemented with butyrate.

D. Application rate (< 1%)

Study	Experimental Total	Experimental Mean	Experimental SD	Control Total	Control Mean	Control SD	SMD	95%-CI	Weight
1	3	4.7	0.7	3	2.5	0.5	2.78	[-0.21; 5.76]	24.0%
2	3	4.3	0.7	3	2.4	0.5	2.48	[-0.28; 5.25]	26.7%
3	3	3.9	0.7	3	3.6	0.6	0.37	[-1.26; 2.01]	49.3%
**Random effects model**	**9**			**9**			**1.51**	**[-0.19; 3.22]**	**100.0%**

Heterogeneity: $I^2 = 31\%$, $\tau^2 = 0.8411$, $p = 0.24$

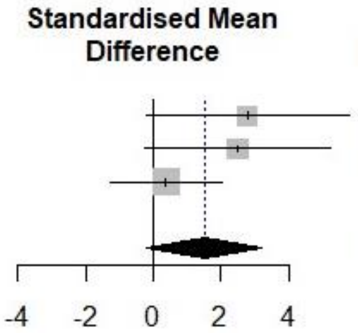

Figure S19. Effect sizes of digestive enzyme in freshwater species supplemented with less than 1% butyrate.

Study	Experimental Total	Experimental Mean	Experimental SD	Control Total	Control Mean	Control SD	SMD	95%-CI	Weight
1	3	1.6	0.4	3	1.2	0.4	0.73	[-1.00; 2.47]	3.4%
2	3	13.2	1.2	3	10.7	1.1	1.73	[-0.51; 3.98]	2.5%
3	3	15.3	1.3	3	15.6	1.3	-0.17	[-1.78; 1.44]	3.7%
4	3	1.5	0.4	3	1.2	0.4	0.64	[-1.06; 2.34]	3.5%
5	3	13.1	1.2	3	10.7	1.1	1.67	[-0.53; 3.88]	2.5%
6	3	15.4	1.3	3	15.6	1.3	-0.12	[-1.72; 1.49]	3.7%
7	3	0.2	0.1	3	0.2	0.1	0.00	[-1.60; 1.60]	3.8%
8	3	5.6	0.8	3	4.5	0.7	1.24	[-0.72; 3.19]	3.0%
9	3	10.8	1.1	3	8.2	1.0	2.03	[-0.41; 4.47]	2.2%
10	3	0.6	0.3	3	0.6	0.3	0.00	[-1.60; 1.60]	3.8%
11	3	83.1	3.0	3	73.8	2.9	2.49	[-0.28; 5.27]	1.8%
12	3	151.4	4.1	3	138.5	3.9	2.57	[-0.26; 5.40]	1.7%
13	3	0.0	0.0	3	0.0	0.0	0.00	[-1.60; 1.60]	3.8%
14	3	0.3	0.2	3	0.3	0.2	0.02	[-1.58; 1.62]	3.8%
15	3	0.5	0.2	3	0.5	0.2	-0.06	[-1.66; 1.55]	3.7%
16	3	0.2	0.1	3	0.5	0.2	-1.32	[-3.31; 0.68]	2.9%
17	3	19.3	1.5	3	17.4	1.4	1.10	[-0.79; 2.98]	3.1%
18	3	35.9	2.0	3	30.3	1.8	2.32	[-0.32; 4.97]	1.9%
19	3	65.0	2.7	3	52.7	2.4	3.85	[0.01; 7.69]	1.0%
20	3	49.3	2.3	3	36.7	2.0	4.62	[0.13; 9.11]	0.8%
21	3	31.3	1.9	3	25.7	1.7	2.54	[-0.27; 5.34]	1.8%
22	3	40.0	2.1	3	38.3	2.1	0.64	[-1.06; 2.34]	3.5%
23	3	66.7	2.7	3	52.7	2.4	4.34	[0.09; 8.59]	0.9%
24	3	48.7	2.3	3	36.7	2.0	4.40	[0.10; 8.70]	0.9%
25	3	26.7	1.7	3	25.7	1.7	0.47	[-1.19; 2.12]	3.6%
26	3	46.7	2.3	3	38.3	2.1	3.06	[-0.15; 6.27]	1.4%
27	3	52.0	2.4	3	48.0	2.3	1.35	[-0.66; 3.37]	2.8%
28	3	51.7	2.4	3	33.3	1.9	6.73	[0.41; 13.05]	0.4%
29	3	34.0	1.9	3	26.0	1.7	3.50	[-0.06; 7.05]	1.2%
30	3	50.7	2.4	3	38.3	2.1	4.43	[0.10; 8.76]	0.9%
31	3	52.0	2.4	3	48.0	2.3	1.35	[-0.66; 3.37]	2.8%
32	3	51.7	2.4	3	33.3	1.9	6.73	[0.41; 13.05]	0.4%
33	3	34.0	1.9	3	26.0	1.7	3.50	[-0.06; 7.05]	1.2%
34	3	50.7	2.4	3	38.3	2.1	4.43	[0.10; 8.76]	0.9%
35	3	51.7	2.4	3	48.0	2.3	1.24	[-0.71; 3.20]	3.0%
36	3	47.7	2.3	3	33.3	1.9	5.39	[0.24; 10.55]	0.6%
37	3	28.7	1.8	3	26.0	1.7	1.22	[-0.73; 3.17]	3.0%
38	3	51.0	2.4	3	38.3	2.1	4.54	[0.12; 8.96]	0.8%
39	3	35.3	2.0	3	31.3	1.9	1.66	[-0.54; 3.86]	2.5%
40	3	34.7	2.0	3	26.7	1.7	3.46	[-0.07; 6.98]	1.2%
41	3	35.7	2.0	3	24.7	1.7	4.79	[0.16; 9.43]	0.8%
42	3	55.0	2.5	3	40.7	2.1	4.96	[0.18; 9.74]	0.7%
43	3	52.7	2.4	3	31.3	1.9	7.88	[0.55; 15.22]	0.3%
44	3	36.7	2.0	3	26.7	1.7	4.25	[0.07; 8.43]	0.9%
45	3	25.7	1.7	3	24.7	1.7	0.48	[-1.18; 2.14]	3.6%
46	3	38.3	2.1	3	40.7	2.1	-0.89	[-2.68; 0.90]	3.3%
**Random effects model**	**138**			**138**			**1.26**	**[0.83; 1.68]**	**100.0%**

Heterogeneity: $I^2 = 38\%$, $\tau^2 = 0.5665$, $p < 0.01$

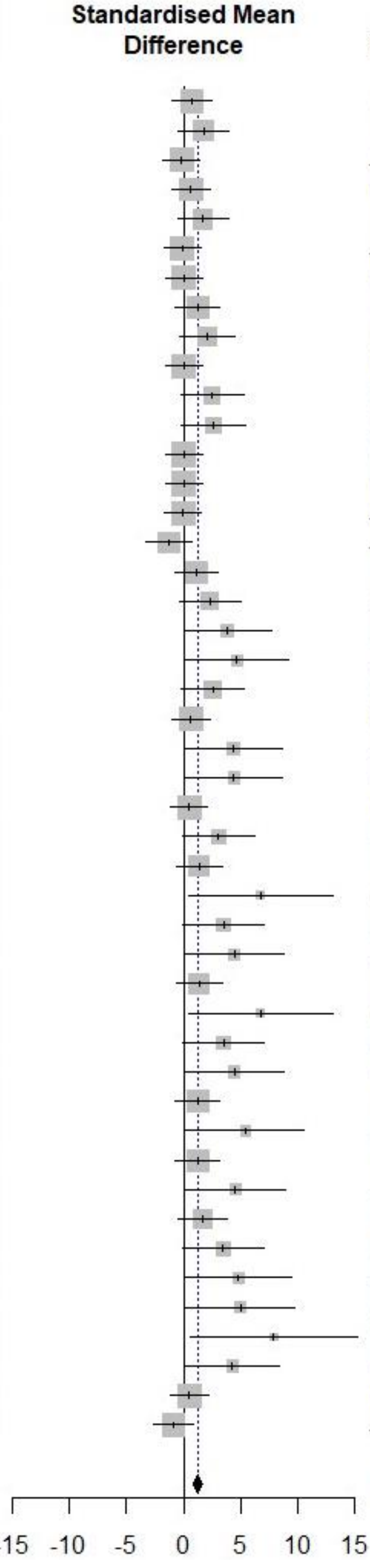

Figure S20. Effect sizes of digestive enzyme in marine species supplemented with less than 1% butyrate.

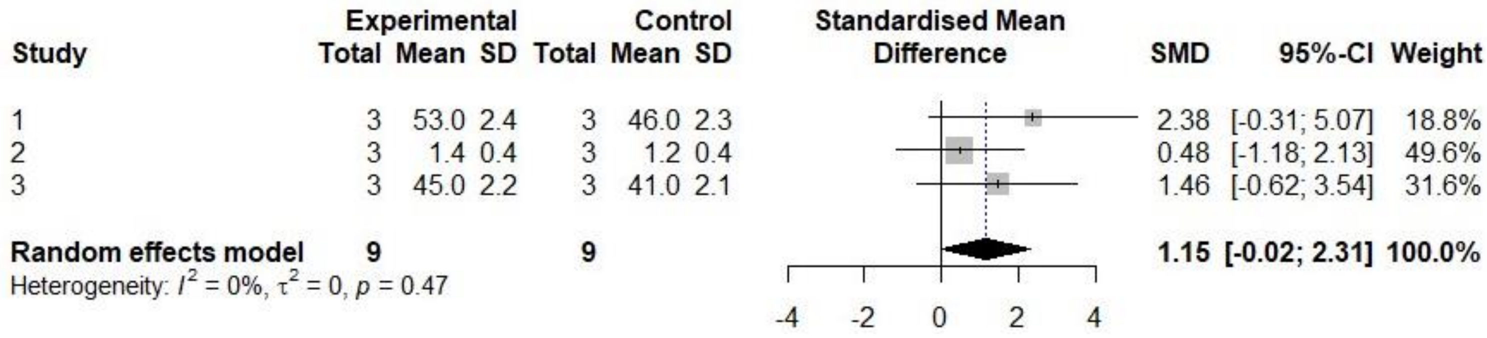

Figure S21. Effect sizes of feed efficiency in freshwater species supplemented with less than 1% butyrate.

Study	Experimental Total	Experimental Mean	Experimental SD	Control Total	Control Mean	Control SD	SMD	95%-CI	Weight
1	3	0.6	0.3	3	0.5	0.2	0.19	[-1.42; 1.80]	5.5%
2	3	27.6	1.8	3	20.6	1.5	3.41	[-0.07; 6.90]	1.2%
3	3	6.1	0.8	3	5.4	0.8	0.70	[-1.02; 2.42]	4.8%
4	3	1.3	0.4	3	1.0	0.3	0.47	[-1.19; 2.12]	5.2%
5	3	0.8	0.3	3	0.7	0.3	0.17	[-1.44; 1.77]	5.6%
6	3	0.7	0.3	3	0.7	0.3	0.06	[-1.55; 1.66]	5.6%
7	3	1.3	0.4	3	1.4	0.4	-0.25	[-1.87; 1.37]	5.5%
8	3	2.1	0.5	3	2.1	0.5	-0.12	[-1.72; 1.49]	5.6%
9	3	1.1	0.4	3	1.1	0.4	0.09	[-1.51; 1.69]	5.6%
10	3	2.0	0.5	3	2.1	0.5	-0.15	[-1.75; 1.46]	5.6%
11	3	1.2	0.4	3	1.1	0.4	0.13	[-1.47; 1.74]	5.6%
12	3	2.0	0.5	3	2.0	0.5	0.02	[-1.58; 1.62]	5.6%
13	3	1.2	0.4	3	1.2	0.4	0.00	[-1.60; 1.60]	5.6%
14	3	2.1	0.5	3	2.0	0.5	0.10	[-1.50; 1.70]	5.6%
15	3	1.2	0.4	3	1.2	0.4	-0.07	[-1.67; 1.54]	5.6%
16	3	2.2	0.5	3	2.4	0.5	-0.22	[-1.83; 1.39]	5.5%
17	3	1.1	0.4	3	1.1	0.3	0.11	[-1.49; 1.72]	5.6%
18	3	2.1	0.5	3	2.4	0.5	-0.43	[-2.08; 1.22]	5.3%
19	3	1.2	0.4	3	1.1	0.3	0.27	[-1.35; 1.89]	5.5%
**Random effects model**	**57**			**57**			**0.09**	**[-0.28; 0.47]**	**100.0%**

Heterogeneity: $I^2 = 0\%$, $\tau^2 = 0$, $p = 1.00$

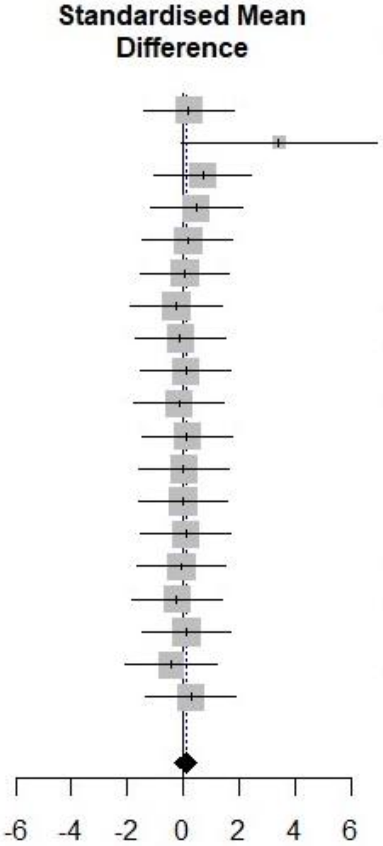

Figure S22. Effect sizes of feed efficiency in marine species supplemented with less than 1% butyrate.

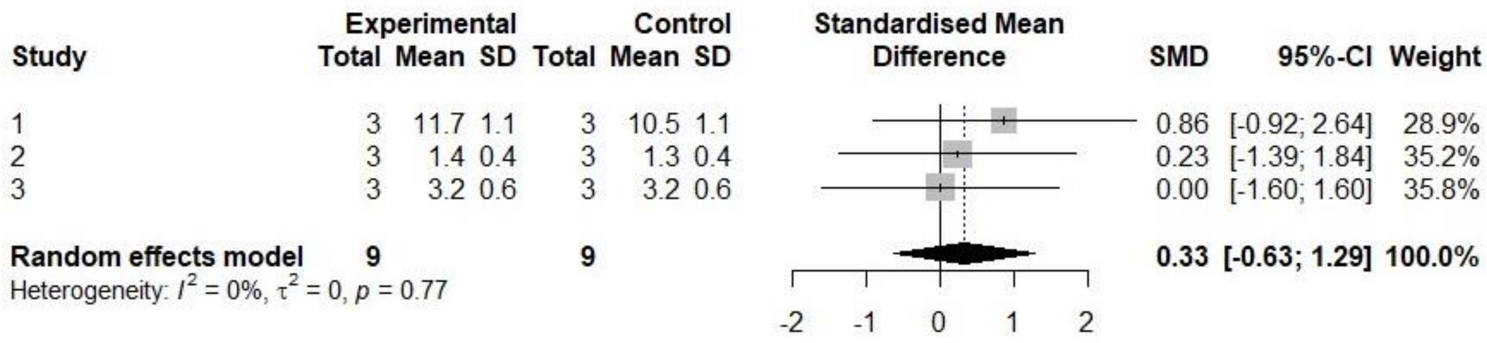

Figure S23. Effect sizes of growth performance in freshwater species supplemented with less than 1% butyrate.

Study	Experimental Total	Experimental Mean	Experimental SD	Control Total	Control Mean	Control SD	SMD	95%-CI	Weight
1	3	10.6	1.1	3	9.3	1.0	0.97	[-0.86; 2.79]	3.6%
2	3	1.2	0.4	3	0.9	0.3	0.60	[-1.09; 2.29]	4.2%
3	3	1176.0	11.4	3	992.0	10.5	13.38	[1.13; 25.63]	0.1%
4	3	92.3	3.2	3	88.7	3.1	0.91	[-0.89; 2.71]	3.7%
5	3	413.2	6.8	3	401.9	6.7	1.34	[-0.67; 3.36]	3.0%
6	3	0.7	0.3	3	0.7	0.3	0.06	[-1.54; 1.66]	4.7%
7	3	65.8	2.7	3	56.7	2.5	2.80	[-0.20; 5.81]	1.3%
8	3	447.3	7.0	3	401.9	6.7	5.28	[0.23; 10.33]	0.5%
9	3	0.8	0.3	3	0.7	0.3	0.22	[-1.39; 1.84]	4.6%
10	3	65.8	2.7	3	56.7	2.5	2.80	[-0.20; 5.81]	1.3%
11	3	8.9	1.0	3	7.6	0.9	1.07	[-0.80; 2.94]	3.4%
12	3	92.0	3.2	3	87.0	3.1	1.26	[-0.71; 3.23]	3.1%
13	3	18.3	1.4	3	17.3	1.4	0.57	[-1.11; 2.25]	4.3%
14	3	13.5	1.2	3	12.5	1.2	0.66	[-1.05; 2.38]	4.1%
15	3	63.0	2.6	3	66.7	2.7	-1.09	[-2.97; 0.79]	3.4%
16	3	19.2	1.5	3	17.3	1.4	1.03	[-0.82; 2.88]	3.5%
17	3	14.2	1.2	3	12.5	1.2	1.12	[-0.78; 3.01]	3.4%
18	3	70.3	2.8	3	66.7	2.7	1.06	[-0.81; 2.92]	3.5%
19	3	18.9	1.4	3	17.6	1.4	0.71	[-1.01; 2.44]	4.0%
20	3	14.0	1.2	3	13.1	1.2	0.58	[-1.10; 2.27]	4.2%
21	3	59.0	2.6	3	60.0	2.6	-0.31	[-1.94; 1.31]	4.6%
22	3	18.7	1.4	3	17.6	1.4	0.63	[-1.07; 2.34]	4.2%
23	3	13.7	1.2	3	13.1	1.2	0.39	[-1.25; 2.03]	4.5%
24	3	67.3	2.7	3	60.0	2.6	2.20	[-0.36; 4.76]	1.8%
25	3	16.9	1.4	3	15.9	1.3	0.57	[-1.11; 2.26]	4.2%
26	3	11.8	1.1	3	10.9	1.1	0.64	[-1.06; 2.34]	4.2%
27	3	62.3	2.6	3	60.7	2.6	0.51	[-1.16; 2.17]	4.3%
28	3	17.6	1.4	3	15.9	1.3	0.98	[-0.85; 2.81]	3.6%
29	3	12.8	1.2	3	10.9	1.1	1.28	[-0.70; 3.26]	3.1%
30	3	68.7	2.8	3	60.7	2.6	2.38	[-0.31; 5.07]	1.7%
**Random effects model**	**90**			**90**			**0.77**	**[0.42; 1.11]**	**100.0%**

Heterogeneity: $I^2 = 0\%$, $\tau^2 < 0.0001$, $p = 0.83$

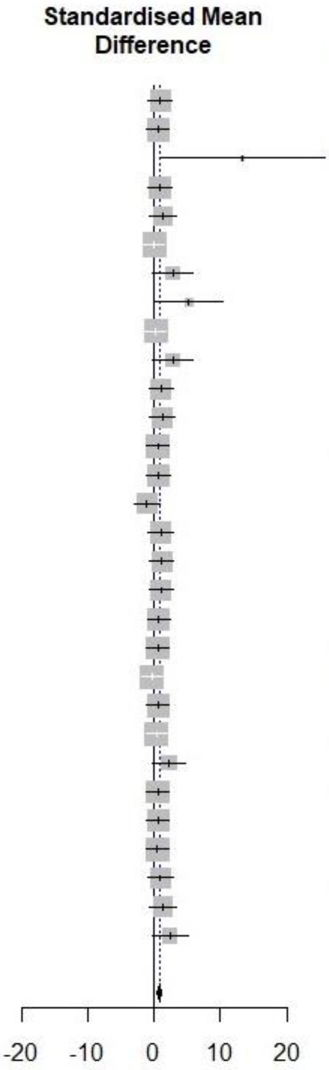

Figure S24. Effect sizes of growth performance in marine species supplemented with less than 1% butyrate.

E. Application rate (1-2%)

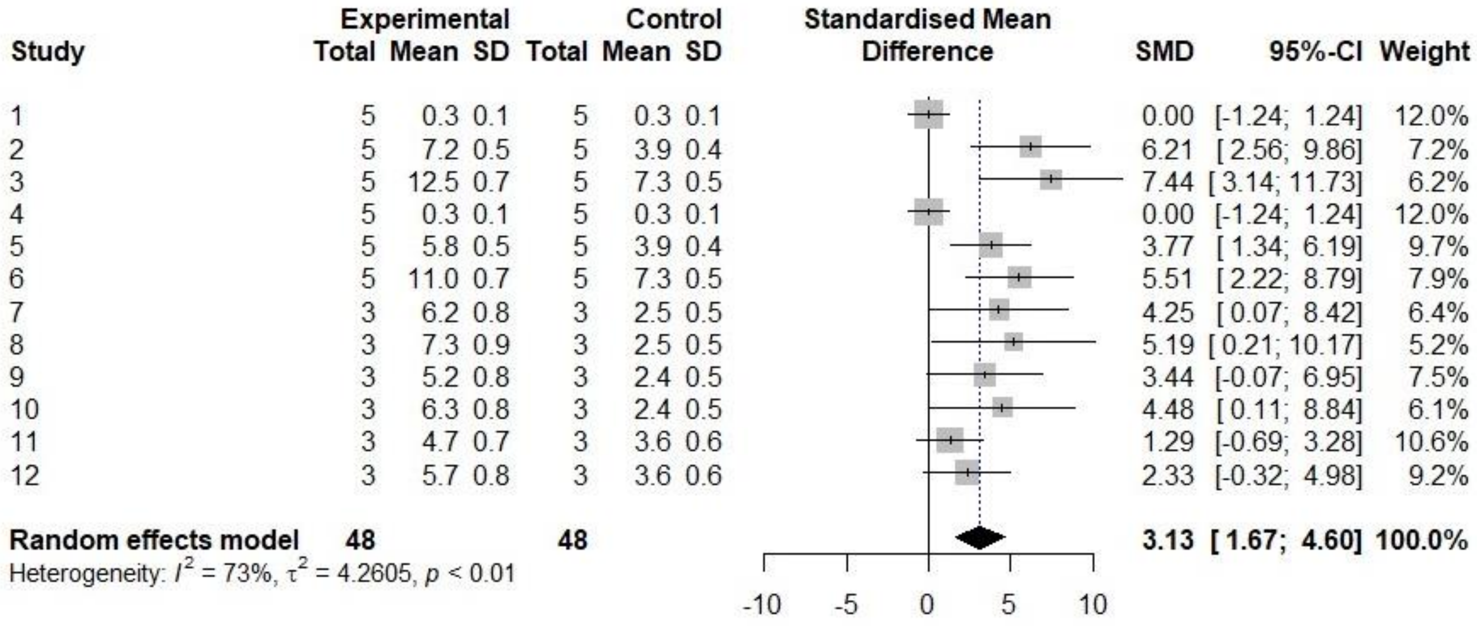

Figure S25. Effect sizes of digestive enzyme in freshwater species supplemented with 1 to 2% butyrate.

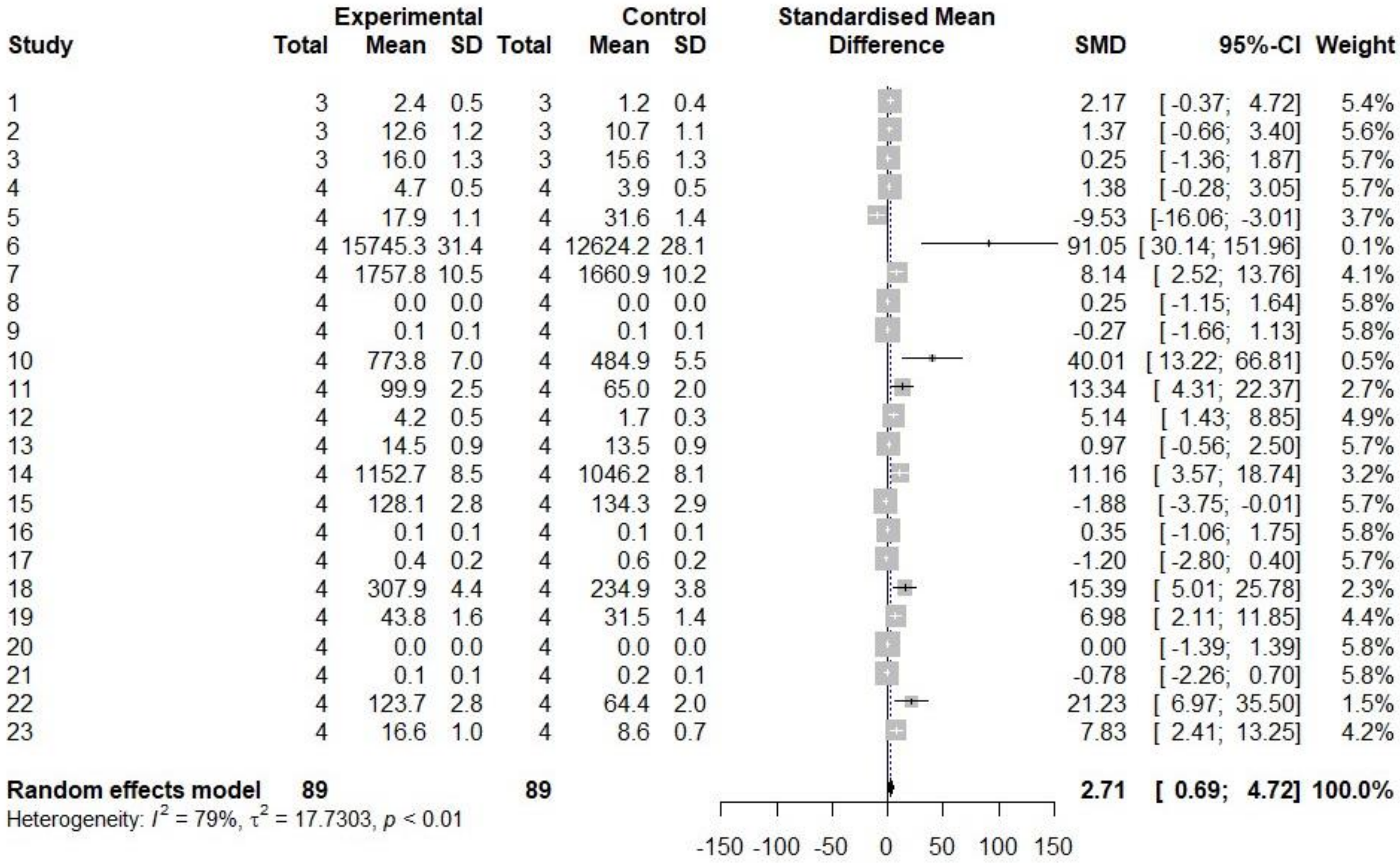

Figure S26. Effect sizes of digestive enzyme in marine species supplemented with 1 to 2% butyrate.

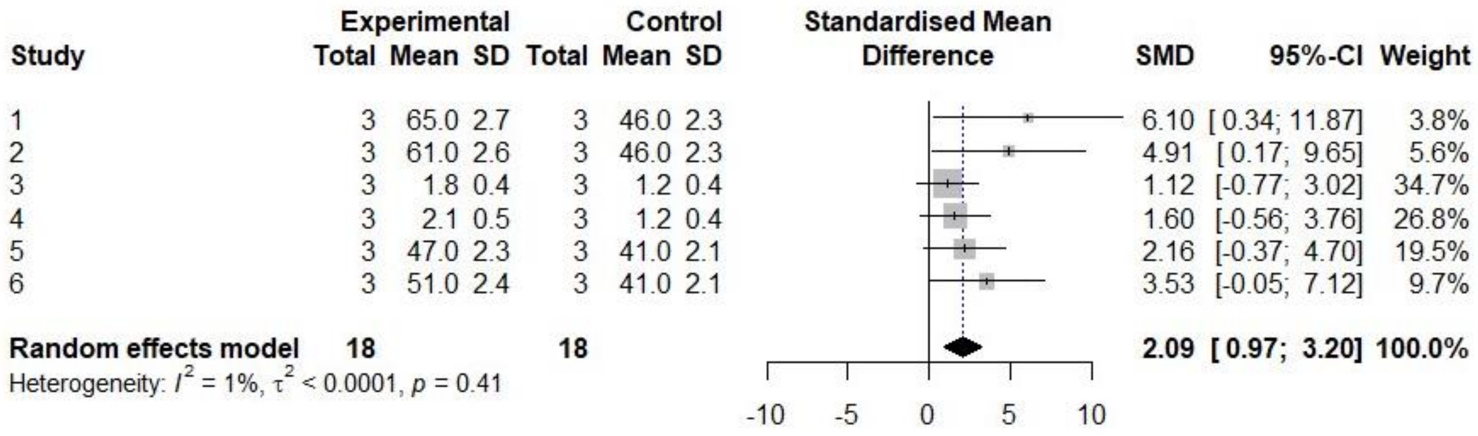

Figure S27. Effect sizes of feed efficiency in freshwater species supplemented with 1 to 2% butyrate.

Study	Experimental Total	Experimental Mean	Experimental SD	Control Total	Control Mean	Control SD	SMD	95%-CI	Weight
1	4	2.2	0.4	4	1.9	0.3	0.73	[-0.74; 2.20]	6.0%
2	4	2.0	0.4	4	1.9	0.3	0.25	[-1.15; 1.64]	6.7%
3	3	4.0	0.7	3	2.8	0.6	1.56	[-0.58; 3.69]	2.9%
4	3	0.6	0.2	3	0.5	0.2	0.07	[-1.54; 1.67]	5.1%
5	3	0.6	0.3	3	0.5	0.2	0.25	[-1.36; 1.87]	5.0%
6	3	24.4	1.6	3	20.6	1.5	1.92	[-0.45; 4.28]	2.3%
7	3	5.8	0.8	3	5.4	0.8	0.40	[-1.24; 2.05]	4.8%
8	3	1.2	0.4	3	1.0	0.3	0.29	[-1.33; 1.92]	5.0%
9	3	28.1	1.8	3	20.6	1.5	3.64	[-0.03; 7.31]	1.0%
10	3	6.0	0.8	3	5.4	0.8	0.60	[-1.09; 2.29]	4.6%
11	3	1.4	0.4	3	1.0	0.3	0.70	[-1.02; 2.42]	4.4%
12	4	0.5	0.2	4	0.4	0.2	0.05	[-1.33; 1.44]	6.8%
13	4	34.7	1.5	4	31.3	1.4	2.06	[0.11; 4.02]	3.4%
14	4	11.8	0.9	4	12.4	0.9	-0.55	[-1.98; 0.88]	6.3%
15	4	1.5	0.3	4	1.6	0.3	-0.17	[-1.56; 1.22]	6.7%
16	3	0.8	0.3	3	0.7	0.3	0.25	[-1.37; 1.86]	5.0%
17	4	1.3	0.3	4	1.4	0.4	-0.23	[-1.62; 1.17]	6.7%
18	4	1.5	0.3	4	2.4	0.4	-2.24	[-4.28; -0.20]	3.1%
19	4	0.1	0.1	4	0.1	0.1	0.34	[-1.07; 1.74]	6.6%
20	4	0.1	0.1	4	0.1	0.1	0.44	[-0.98; 1.86]	6.5%
21	4	38.0	1.5	4	27.0	1.3	6.70	[2.01; 11.40]	0.6%
22	4	41.0	1.6	4	27.0	1.3	8.34	[2.59; 14.09]	0.4%
**Random effects model**	**78**			**78**			**0.37**	**[0.01; 0.73]**	**100.0%**

Heterogeneity: $I^2 = 37\%$, $\tau^2 < 0.0001$, $p = 0.04$

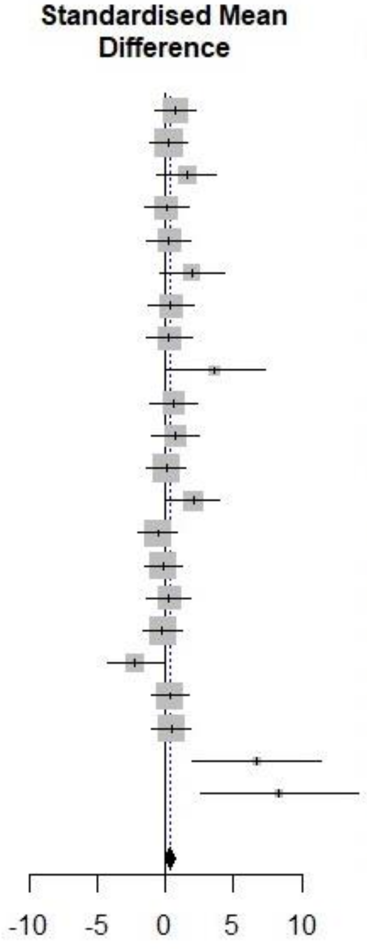

Figure S28. Effect sizes of feed efficiency in marine species supplemented with 1 to 2% butyrate.

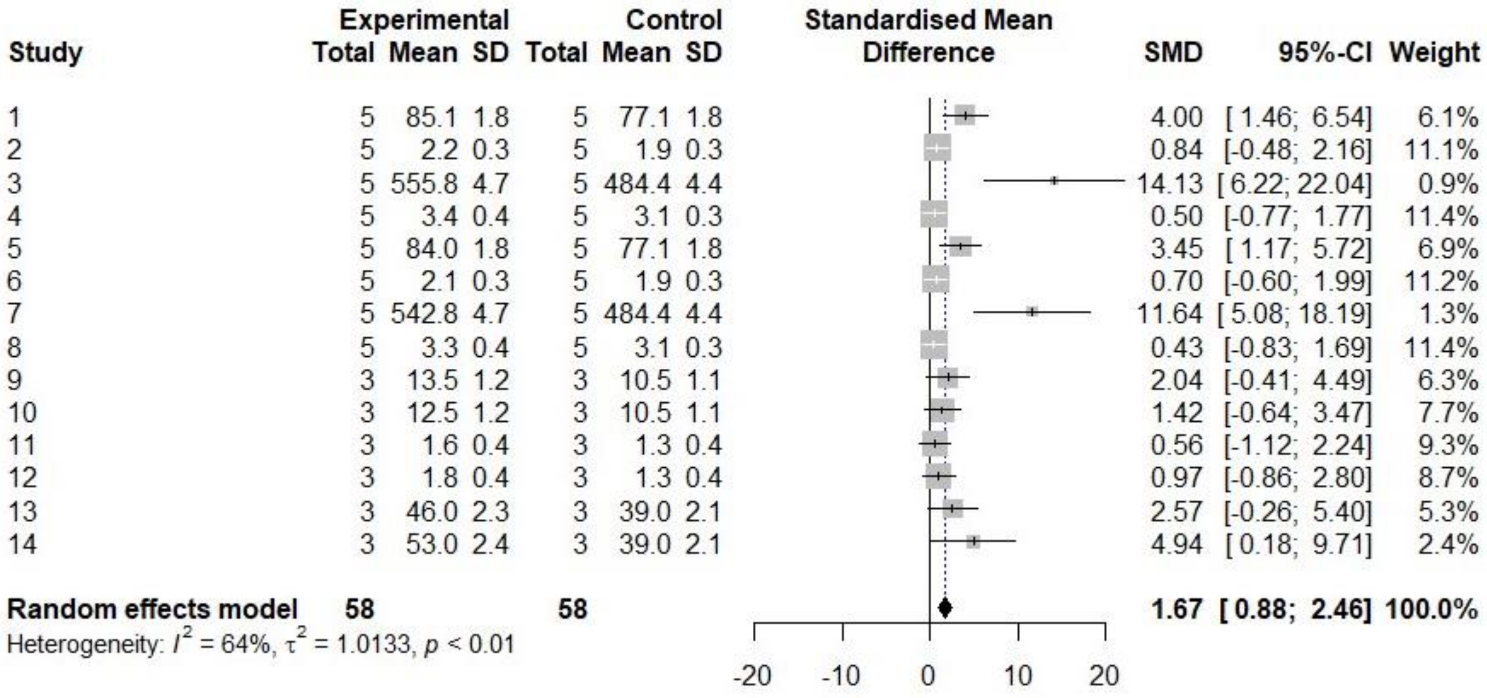

Figure S29. Effect sizes of growth performance in freshwater species supplemented with 1 to 2% butyrate.

Study	Experimental Total	Experimental Mean	Experimental SD	Control Total	Control Mean	Control SD	SMD	95%-CI	Weight
1	4	100.2	2.5	4	100.0	2.5	0.09	[-1.30; 1.47]	3.1%
2	4	83.6	2.3	4	59.3	1.9	9.97	[3.16; 16.78]	1.3%
3	4	80.9	2.2	4	49.4	1.8	13.54	[4.38; 22.70]	0.9%
4	4	81.2	2.2	4	49.7	1.8	13.54	[4.38; 22.70]	0.9%
5	4	80.9	2.2	4	49.4	1.8	13.54	[4.38; 22.70]	0.9%
6	4	99.8	2.5	4	100.0	2.5	-0.09	[-1.47; 1.30]	3.1%
7	4	81.4	2.3	4	59.3	1.9	9.12	[2.86; 15.37]	1.4%
8	4	76.5	2.2	4	49.4	1.8	11.82	[3.79; 19.84]	1.1%
9	4	76.5	2.2	4	49.7	1.8	11.71	[3.76; 19.66]	1.1%
10	4	76.0	2.2	4	49.4	1.8	11.63	[3.73; 19.53]	1.1%
11	4	12.1	0.9	4	12.6	0.9	-0.46	[-1.88; 0.96]	3.0%
12	4	1.7	0.3	4	1.8	0.3	-0.26	[-1.66; 1.14]	3.0%
13	4	12.3	0.9	4	12.6	0.9	-0.23	[-1.62; 1.17]	3.0%
14	4	1.8	0.3	4	1.8	0.3	-0.18	[-1.57; 1.21]	3.1%
15	3	10.2	1.1	3	9.3	1.0	0.71	[-1.02; 2.44]	3.0%
16	3	1.1	0.4	3	0.9	0.3	0.49	[-1.17; 2.15]	3.0%
17	3	1100.0	11.1	3	992.0	10.5	7.99	[0.56; 15.42]	1.2%
18	3	89.3	3.1	3	88.7	3.1	0.15	[-1.45; 1.76]	3.0%
19	3	10.8	1.1	3	9.3	1.0	1.13	[-0.77; 3.03]	2.9%
20	3	1.2	0.4	3	0.9	0.3	0.69	[-1.03; 2.41]	3.0%
21	3	1237.0	11.7	3	992.0	10.5	17.57	[1.54; 33.60]	0.4%
22	3	93.0	3.2	3	88.7	3.1	1.08	[-0.80; 2.96]	2.9%
23	4	13.3	0.9	4	13.3	0.9	0.01	[-1.38; 1.40]	3.1%
24	4	1.6	0.3	4	1.6	0.3	0.03	[-1.36; 1.41]	3.1%
25	4	81.6	2.3	4	76.5	2.2	2.00	[0.08; 3.93]	2.9%
26	4	2.7	0.4	4	2.5	0.4	0.36	[-1.04; 1.77]	3.0%
27	3	433.2	6.9	3	401.9	6.7	3.68	[-0.03; 7.38]	2.3%
28	3	0.8	0.3	3	0.7	0.3	0.17	[-1.44; 1.78]	3.0%
29	3	67.5	2.7	3	56.7	2.5	3.29	[-0.10; 6.68]	2.4%
30	4	25.8	1.3	4	19.3	1.1	4.75	[1.29; 8.22]	2.3%
31	4	28.3	1.3	4	11.6	0.8	13.00	[4.20; 21.80]	0.9%
32	4	28.4	1.3	4	19.3	1.1	6.48	[1.93; 11.02]	1.9%
33	4	28.3	1.3	4	11.6	0.8	13.00	[4.20; 21.80]	0.9%
34	4	22.0	1.2	4	18.0	1.1	3.11	[0.61; 5.61]	2.7%
35	4	22.0	1.2	4	18.0	1.1	3.11	[0.61; 5.61]	2.7%
36	4	3.6	0.5	4	2.9	0.4	1.35	[-0.30; 3.00]	3.0%
37	4	3.9	0.5	4	2.9	0.4	1.88	[0.01; 3.76]	2.9%
38	4	98.0	2.5	4	70.0	2.1	10.63	[3.39; 17.87]	1.2%
39	4	98.0	2.5	4	65.0	2.0	12.70	[4.10; 21.31]	1.0%
40	4	95.0	2.4	4	70.0	2.1	9.56	[3.02; 16.10]	1.4%
41	4	90.0	2.4	4	65.0	2.0	9.86	[3.12; 16.60]	1.3%
42	4	0.5	0.2	4	0.4	0.2	0.51	[-0.92; 1.94]	3.0%
43	4	2.9	0.4	4	2.2	0.4	1.52	[-0.20; 3.23]	3.0%
44	4	0.5	0.2	4	0.4	0.2	0.51	[-0.92; 1.94]	3.0%
45	4	3.2	0.4	4	2.2	0.4	2.11	[0.13; 4.09]	2.9%
**Random effects model**	**169**			**169**			**2.87**	**[1.86; 3.87]**	**100.0%**

Heterogeneity: $I^2 = 71\%$, $\tau^2 = 8.1418$, $p < 0.01$

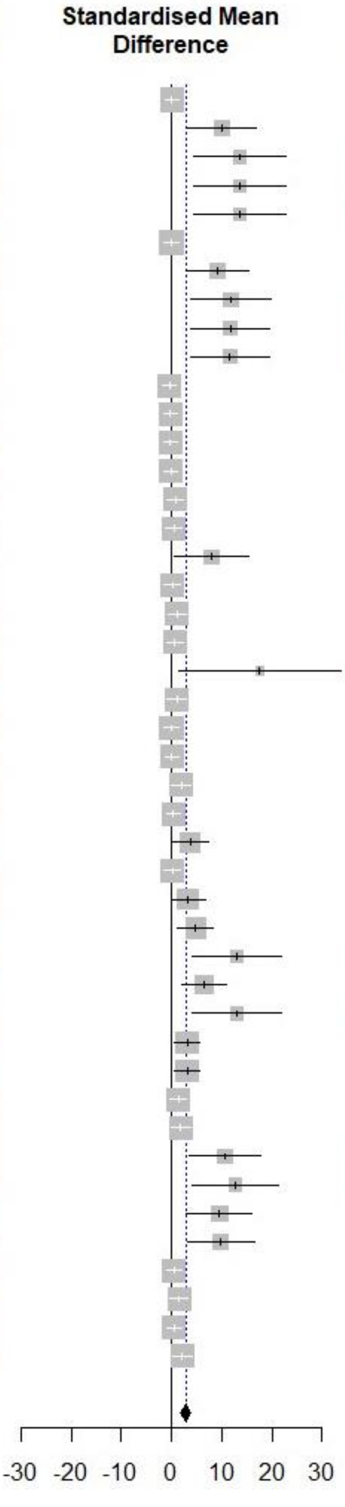

Figure S30. Effect sizes of growth performance in marine species supplemented with 1 to 2% butyrate.

F. Application rate (> 2%)

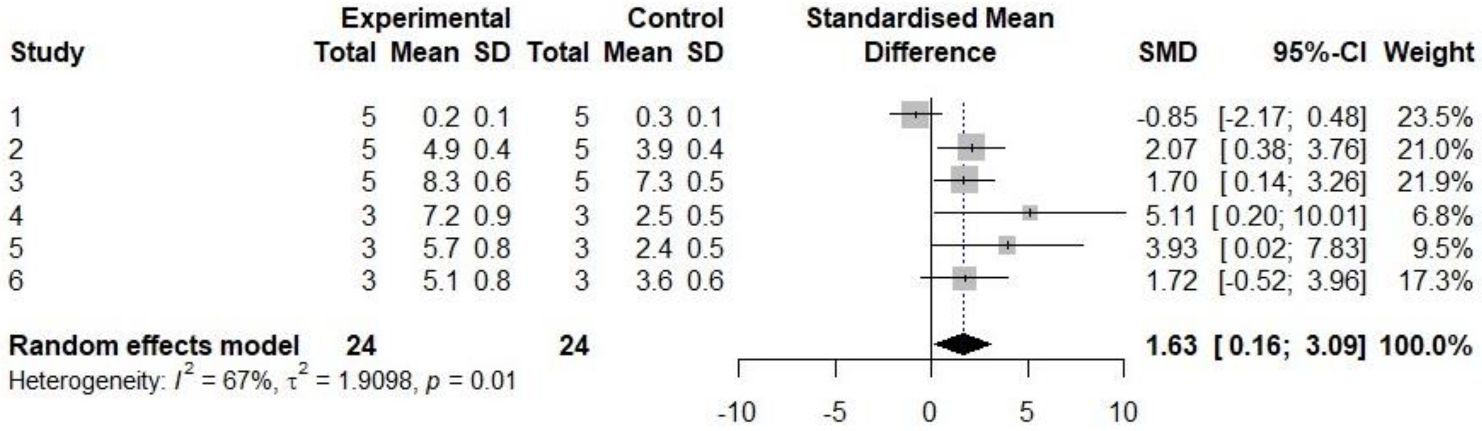

Figure S31. Effect sizes of digestive enzyme in freshwater species supplemented with more than 2% butyrate.

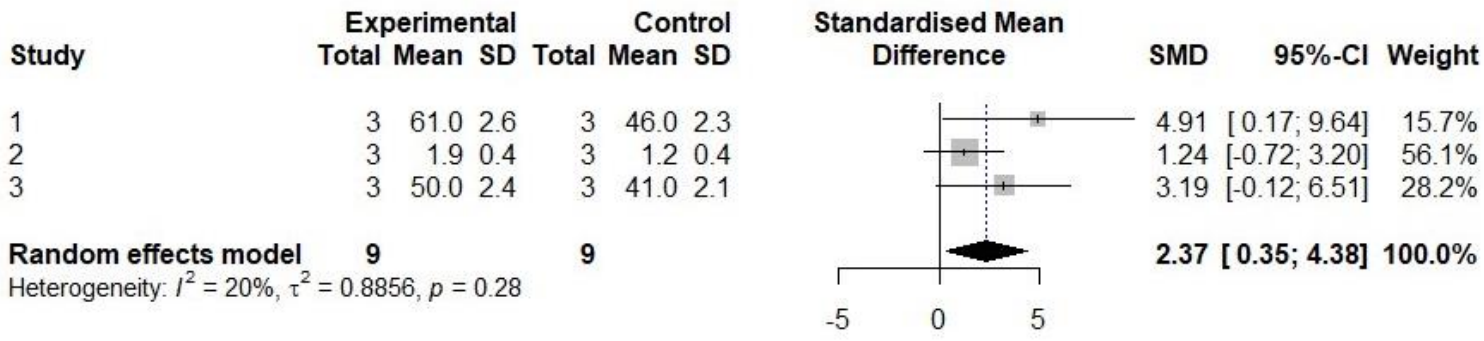

Figure S32. Effect sizes of feed efficiency in freshwater species supplemented with more than 2% butyrate.

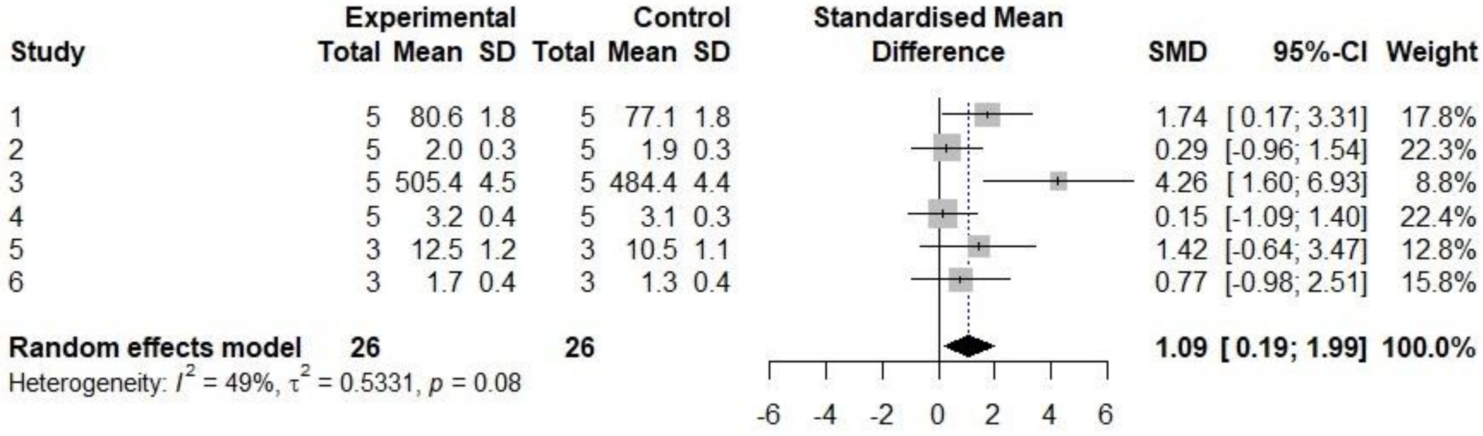

Figure S33. Effect sizes of growth performance in freshwater species supplemented with more than 2% butyrate.
